# Updated A(H1N1)pdm09 influenza virus ferret infection model permits refined antiviral assessment using aerosol inhalation challenge

**DOI:** 10.1371/journal.ppat.1014604

**Published:** 2026-09-09

**Authors:** Harry L. Stannard, Nicole Brock, Xiangjie Sun, Joanna A. Pulit-Penaloza, Troy J. Kieran, Ashwin Muraleetharan, Clyde Dapat, Smitha Rose Georgy, Taronna R. Maines, Ian G. Barr, Patrick C. Reading, Saira Hussain, Jessica A. Belser

**Affiliations:** 1 Victorian Infectious Diseases Reference Laboratory, at The Peter Doherty Institute for Infection and Immunity, Melbourne, Victoria, Australia; 2 Department of Microbiology and Immunology, University of Melbourne, at the Peter Doherty Institute for Infection and Immunity, Melbourne, Victoria, Australia; 3 Influenza Division, National Center for Immunization and Respiratory Diseases, Centers for Disease Control and Prevention, Atlanta, Georgia, United States of America; 4 Section of Anatomic Pathology, Melbourne Veterinary School, Faculty of Science, The University of Melbourne, Melbourne, Victoria, Australia; University of North Carolina at Chapel Hill, UNITED STATES OF AMERICA

## Abstract

Seasonal influenza viruses continue to pose a significant threat to human health. As influenza viruses exhibit sustained genetic drift, it is imperative that animal studies utilize challenge strains that reflect contemporary, currently circulating viruses when evaluating pathogenicity, viral tropism, transmissibility, and antiviral sensitivity to better inform public health responses. Ferrets are considered the gold-standard small animal model for assessing currently circulating influenza viruses. Seasonal influenza A(H1N1)pdm09 viruses replicate well in both the upper and lower respiratory tract of ferrets, providing an important model for developing improved vaccination and therapeutic strategies; however, many of these studies have relied on a 2009 virus isolate. Utilising representative influenza A(H1N1)pdm09 virus strains from 2009 to 2022, we explored virus replication kinetics and lung pathogenesis in ferrets following intranasal inoculation with these contemporary strains. Our results revealed strain specific differences, with greater lung viral loads and pathogenesis following inoculation with A/Sydney/5/2021 compared to other strains. Efficient transmissibility of A/Sydney/5/2021 virus to naïve recipients was also observed following both contact and airborne exposure to infected donor ferrets. To refine this updated model, we performed side-by-side evaluation of oseltamivir antiviral efficacy following traditional intranasal or aerosol inhalation influenza challenges. Pre-treatment with oseltamivir demonstrated greater reductions in viral shedding from the upper respiratory tract than post-infection treatment of ferrets infected by aerosol inhalation, while the intranasal route showed reduced oseltamivir efficacy independent of the timing of antiviral treatment. These findings provide the basis for using an updated A(H1N1)pdm09 challenge virus for ferret studies as an alternative to the commonly used, but now less relevant 2009 early pandemic viruses. It also highlights how different methods of virus inoculation can influence outcomes of ferret antiviral studies, with an aerosol challenge model able to demonstrate differences between therapeutic and pre-treatment schedules, which were not apparent with an intranasal challenge model.

## Author summary

Small mammalian models serve a critical role for assessing the efficacy of existing and novel antiviral drugs against influenza A virus. However, drug efficacy studies in the ferret model frequently use an A(H1N1)pdm09 virus challenge strain that was isolated over 15 years ago, and inoculate animals in a way that does not reflect how humans are typically exposed to influenza virus. In this study, we first examined a panel of contemporary A(H1N1)pdm09 viruses and identified a strain from 2021 that is well-suited to serve as an updated challenge virus for these studies. Using this 2021 strain, we performed a head-to-head comparison of two inoculation routes, and found that the antiviral effects of oseltamivir in ferrets were more apparent early after infection following an aerosol inhalation-based inoculation method compared to the standard intranasal inoculation route. Collectively, these findings support that continued refinement of preclinical mammalian models used for antiviral drug assessments can lead to higher relevance and reliability of future studies aiming to explore anti-influenza interventions.

## Introduction

Influenza virus infections cause a significant global burden on human health. Seasonal influenza epidemics may cause up to 1 billion infections and 650,000 deaths worldwide each year [1,2]. In combination with annual vaccines to seasonal influenza virus, antiviral drugs are an important tool to reduce the disease burden in infected patients. The antiviral drugs currently available include four neuraminidase inhibitors (NAIs), namely oseltamivir (OST), zanamivir, peramivir and laninamivir (only licensed in Japan), and the polymerase acidic (PA) endonuclease inhibitor, baloxavir marboxil. However, antiviral resistance caused by several possible mutations have been observed in influenza viruses following treatment with all available antivirals, some of which have spread globally [3], although current rates of resistance are low [4]. The threat of widespread resistance to currently available antivirals, as well as the opportunity to better treat or prevent influenza disease, encourages the development of new anti-influenza drugs with novel mechanisms of action.

Ferrets are considered the gold standard small mammalian model for influenza virus studies, as ferrets are readily infected without prior host-virus adaptation, and display comparable clinical signs and patterns of virus tropism as humans (due to similar sialic acid receptor distribution and anatomy of the respiratory tract) [5,6]. Given the association between lower respiratory tract (LRT) infection and severe disease in humans across the three seasonal influenza virus types/subtypes, challenge strains that replicate productively in both the upper respiratory tract (URT) and LRT in ferrets are particularly useful. Ferret studies have revealed influenza A(H1N1)pdm09 viruses replicate in both URT and LRT tissues [7,8], unlike many A(H3N2) [9–11] and B/Victoria lineage [12] viruses, which replicate poorly in the LRT. It is probable for this reason that the A(H1N1)pdm09 virus infection model has become the most widely utilised antiviral ferret model for assessing a reduction in viral load and clinical disease from novel therapeutics [13–19]. For example, the pre-clinical development of Molnupiravir, a nucleoside analogue developed against influenza, relied mostly on the A(H1N1)pdm09 ferret challenge model [20,21].

While all the above cited antiviral studies in ferrets have used a virus isolated from the initial pandemic in 2009, circulating influenza A(H1N1)pdm09 viruses have accumulated many genetic changes since this time, particularly in the hemagglutinin (HA) and neuraminidase (NA) genes, including changes associated with increased clinical disease severity [22–26]. Notably, the HA-D222G/N change is over-represented in fatal cases of influenza A(H1N1)pdm09 infection and linked to increased risk of lung infection [27,28]. Additionally, during the 2015–16 influenza season in Taiwan, influenza A(H1N1)pdm09 viruses from clade 6B.2 were associated with more disease symptoms and likelihood of pneumonia, than patients infected with the co-circulating clade 6B.1 viruses [29].

Genetic drift to evade vaccine or prior infection immunity within influenza A subtypes is well defined [30–32]. Use of the naïve ferret model permits assessments of the impact of virus evolution on these phenotypic parameters without being confounded by the complex immunological history present in the human population. However, despite the potential differences in clinical outcome following infection with drifted influenza viruses, assessments of viral fitness, disease severity and transmissibility with contemporary seasonal influenza viruses in mammals are rarely assessed.

Most ferret influenza virus challenge models typically utilize intranasal liquid instillation of a high viral load to establish infection (hereafter referred to as intranasal inoculation), due to the resultant high uniformity of infection and reproducibility of this experimental procedure [5]. However, this does not represent a physiologically relevant viral load of exposure or delivery mode. While the predominant route of transmission in humans remains poorly characterized [33], seasonal influenza infections are likely spread most efficiently by airborne inhalation although direct or indirect contact with contaminated surfaces may also play a role. Prior studies seeking to emulate anti-influenza interventions in ferrets infected by more physiologically relevant means have generally utilised intranasally inoculated donor ferrets to infect contact recipient animals [16,34–38]. However, this approach does not permit quantification of the viral load, nor does it allow consistency of inoculation between replicates, or timing of infection, and can exhibit variable kinetics once infection is established [39]. Prior studies have used a low dose intranasal inoculum of seasonal influenza A virus, which may permit a course of disease that more closely emulates that of human viral shedding kinetics [40–42], and exhibit greater sensitivity to assess antiviral efficacy, than ferrets challenged with high viral doses [43–45]. Recent methodological advancements have now enabled controlled aerosol inhalation exposure as an inoculation route for ferret studies, which recapitulates more closely the ferret-to-ferret airborne exposure [33,46], while reducing the number of ferrets required for experiments and also allowing better control over the viral inoculum exposure dose and the timing of infection.

Despite the advantages of utilizing a physiologically relevant exposure route, limited studies involving ferrets inoculated with seasonal influenza A viruses via the aerosol inhalation route have been performed, likely due to the cost and complexity of aerosol delivery devices. Where aerosol exposure has been implemented, differences in infection dynamics (including magnitude, timing and spread of infectious virus detection throughout the respiratory tract) were observed when compared to intranasal inoculation [33,47]. Given the closer biological relevance and previously observed differences from intranasal inoculation, aerosol-based inoculation approaches may serve as a more valuable tool to assess the efficacy of anti-influenza agents in ferrets [48], but these studies have not been performed to date with the ferret aerosol inhalation model.

The present study compared the replication and pathogenesis of five influenza A(H1N1)pdm09 viruses isolated from 2009 to 2022, selected by the WHO influenza vaccine composition advisory committee for inclusion in annual vaccines from 2009 to 2025, and identified a contemporary candidate virus for subsequent use in antiviral assessments. We then evaluated the unique utility of an aerosol exposure ferret model for assessing OST antiviral efficacy alongside the traditional intranasal route, demonstrating advantages of employing a low-dose, more physiologically relevant influenza challenge.

## Results

### Strain-specific influenza A(H1N1)pdm09 virus replication in the respiratory tract of ferrets

Viruses isolated from 2009 to 2022 were selected from WHO Southern Hemisphere vaccine composition recommended strains to represent significant evolutionary changes within the influenza A(H1N1)pdm09 virus subtype. The five viruses selected included A/California/07/2009 as an early pandemic reference virus, A/Michigan/45/2015 (Clade 6B.1, 17 HA amino acid (AA) substitutions from A/California/07/2009), A/Victoria/2570/2019 (Clade 6B.1A.5a.2, 25 HA AA changes), A/Sydney/5/2021 (Clade 6B.1A.5a.2a, 32 HA AA changes), and A/Victoria/4897/2022 (Clade 6B.1A.5a.2a.1, 35 HA AA changes), as shown on an influenza HA phylogenetic tree (S1 Fig). Across all eight gene segments, from A/California/07/2009 to A/Victoria/4897/2022, 136 AA changes were observed, highlighting genetic drift both within and outside of the HA gene (S1 Table). All viruses used were cell-propagated to avoid egg-adapted mutations.

To determine if A(H1N1)pdm09 viruses exhibiting evolutionary changes differed in their replicative fitness throughout the mammalian respiratory tract, we inoculated ferrets with 5 log_10_TCID_50_ of each virus in 500µL intranasally. Replicative fitness was assessed by titration of daily nasal washes (NW), and URT and LRT respiratory tissues harvested from inoculated animals at both day three and day five post-inoculation (p.i.).

**Fig 1:**
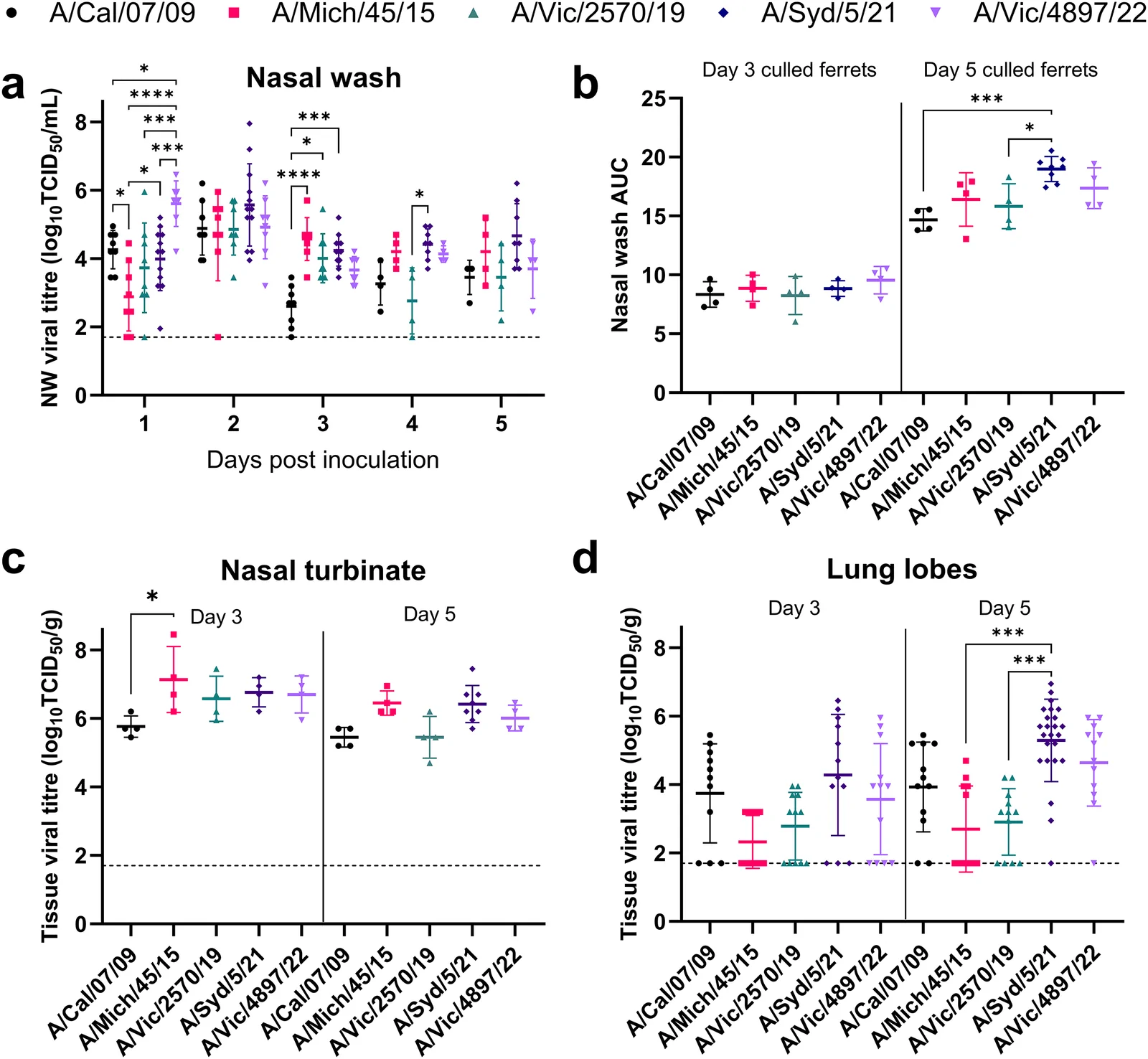
Influenza A(H1N1)pdm09 virus replication in the respiratory tract of ferrets. Ferrets were inoculated by intranasal inoculation with 5 log_10_TCID_50_/500µL of A/California/07/2009, A/Michigan/45/2015, A/Victoria/2570/2019, A/Sydney/5/2021, or A/Victoria/4897/2022 virus. At day three (circles) or five (squares) p.i., respiratory tract tissues were collected for virus titration assay. (a) Daily nasal wash (NW) samples were analysed by TCID_50_ assay. (b) Area under the curve was determined from the NW titre of individual ferrets over time, separated into two groups per virus for ferrets culled at day three or day five p.i. Viral titres from (c) nasal turbinate homogenates and (d) lung lobe homogenates (three lobes per ferret, shown individually) are reported. Dots represent individual specimens collected from four ferrets (eight for A/Sydney/5/2021 day five), with lines and error bars reporting the mean and SD, compared across each group by (a) two-way ANOVA, (b,c) ordinary one-way ANOVA Tukey post-hoc test, or (d) Kruskal-Wallis Dunn’s test (* p < 0.05, ** p < 0.01, *** p < 0.001, **** p < 0.0001).

NW titres indicated some differences between viruses, however mean peaks of viral shedding for ferrets infected with each virus were comparable, ranging between 4.7 and 5.6 log_10_TCID_50_/mL. Peak shedding occurred at day two p.i. for all viruses, except A/Victora/4897/2022 at day one p.i., resulting in statistically significant differences at this initial timepoint (Fig 1a). Area under the curve (AUC) analysis of NW titres from each animal highlighted greater total shedding over five days following inoculation with A/Sydney/5/2021 virus compared to all other viruses tested, with this difference reaching statistical significance against both A/California/07/2009 and A/Victoria/2570/2019 viruses (Fig 1b). Nasal turbinate tissue mean titres across all viruses ranged from 5.5 to 7.1 log_10_TCID_50_/g, with few statistically significant differences observed at either day three or five p.i. (Fig 1c).

Among all viruses tested, A/Sydney/5/2021 virus replicated to the highest mean viral titre in homogenized lung lobes collected either day three or five p.i. (4.3 and 5.3 log_10_TCID_50_/g, respectively), with statistically significant differences observed to A/Michigan/45/2015 and A/Victoria/2570/2019 viruses day five p.i. (Fig 1d).

All ferrets inoculated with A(H1N1)pdm09 viruses exhibited peak weight loss of 5 – 14%, with the exception of four A/Michigan/45/2015 and one A/Victoria/2570/2019 virus-inoculated ferrets, which lost less than 4% body weight. Notably, A/Sydney/5/2021 inoculations resulted in significantly more weight loss than A/California/07/2009 at day five p.i. (-9.2% and -2.0% mean weight change, respectively). The AUC also revealed greater overall weight loss in A/Sydney/5/2021 virus-inoculated ferrets than ferrets inoculated with A/California/07/2009, A/Michigan/45/2015, or A/Victoria/2570/2019. Of note, body temperature change analysis did not reveal any obvious trends, due to considerable variation between animals (S2 Fig).

Collectively, these findings support that while all A(H1N1)pdm09 viruses tested were capable of robust and productive replication throughout the ferret respiratory tract, A/Sydney/5/2021 virus inoculation resulted in the highest levels of morbidity and viral burden during the acute phase of infection.

### Strain-specific influenza A(H1N1)pdm09 virus-induced lung histopathology in ferrets

**Fig 2:**
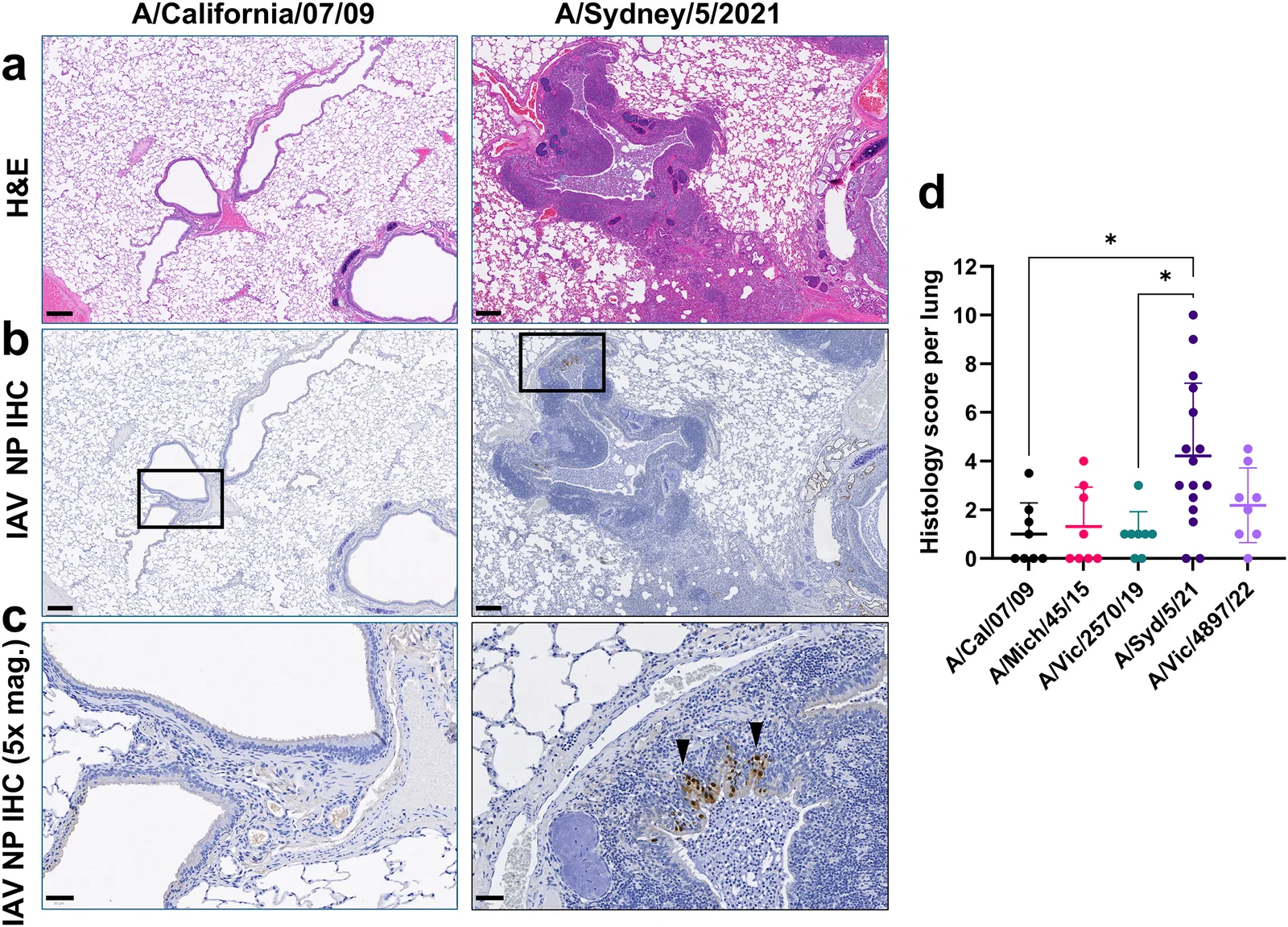
Influenza A(H1N1)pdm09 virus induced histopathological changes in lung lobes of ferrets. Ferrets infected with 5 log_10_TCID_50_/500µL of A/California/07/2009, A/Michigan/45/2015, A/Victoria/2570/2019, A/Sydney/5/2021, or A/Victoria/4897/2022 were culled at day five post intranasal inoculation. Intact trachea and lungs were removed, inflated with formalin for histopathological analysis. (a) Representative slides from ferret lung infected with A/California/07/2009 or A/Sydney/5/2021 were stained by hematoxylin and eosin, or (b) immunohistochemistry (IHC) with influenza A virus nucleoprotein (IAV NP) antibody, and (c) higher magnification scans (black box of b) revealed sites of virus localisation (black arrows). Black scale markers indicate 250 µm (panel a, b) or 50 µm (panel c). (d) Total cumulative histopathology score for bronchiolar and alveolar inflammation (four different categories, each scored 0-3) from the entire lung section (two per animal). Dots represent individual specimens collected from four ferrets (eight for A/Sydney/5/2021 day five), with lines and error bars reporting the mean and SD, compared across each group by Kruskal-Wallis with Dunn’s multiple comparison (*p < 0.05).

We next assessed if differences observed in viral replication in the lungs of ferrets at day five p.i. was associated with increased histopathological changes in this tissue. Analysis of hematoxylin and eosin-stained lung lobes from ferrets inoculated with A(H1N1)pdm09 viruses revealed varied levels of inflammation including necrosis of bronchial glands, perivascular oedema extending to the alveoli, attenuation or degenerative changes in the bronchiolar epithelium (Fig 2). In agreement with viral titre data, A/Sydney/5/2021 virus-inoculated ferrets had a higher mean histopathology score for lung lobes than the other challenge viruses evaluated (A/California/07/2009; 1.0, A/Michigan/45/2015; 1.3, A/Victoria/2570/2019; 1, A/Sydney/5/2021; 4.2, A/Victoria/4897/2022; 2.2) with sites of lung tissue damage associated with virus, detected by immunohistochemistry (IHC; Fig 2c, black arrows and S3 Fig). All viruses were detected to varying levels in ferret lung by IHC, which largely correlated to histology scores (Fig 2d). Taken together, A/Sydney/5/2021 was identified as a contemporary A(H1N1)pdm09 virus associated with more severe disease in the intranasal challenge ferret model, and was therefore selected for subsequent characterization as a candidate strain for therapeutic challenge studies.

### Efficient transmissibility of influenza A(H1N1)pdm09 A/Sydney/5/2021 virus between ferrets

**Fig 3:**
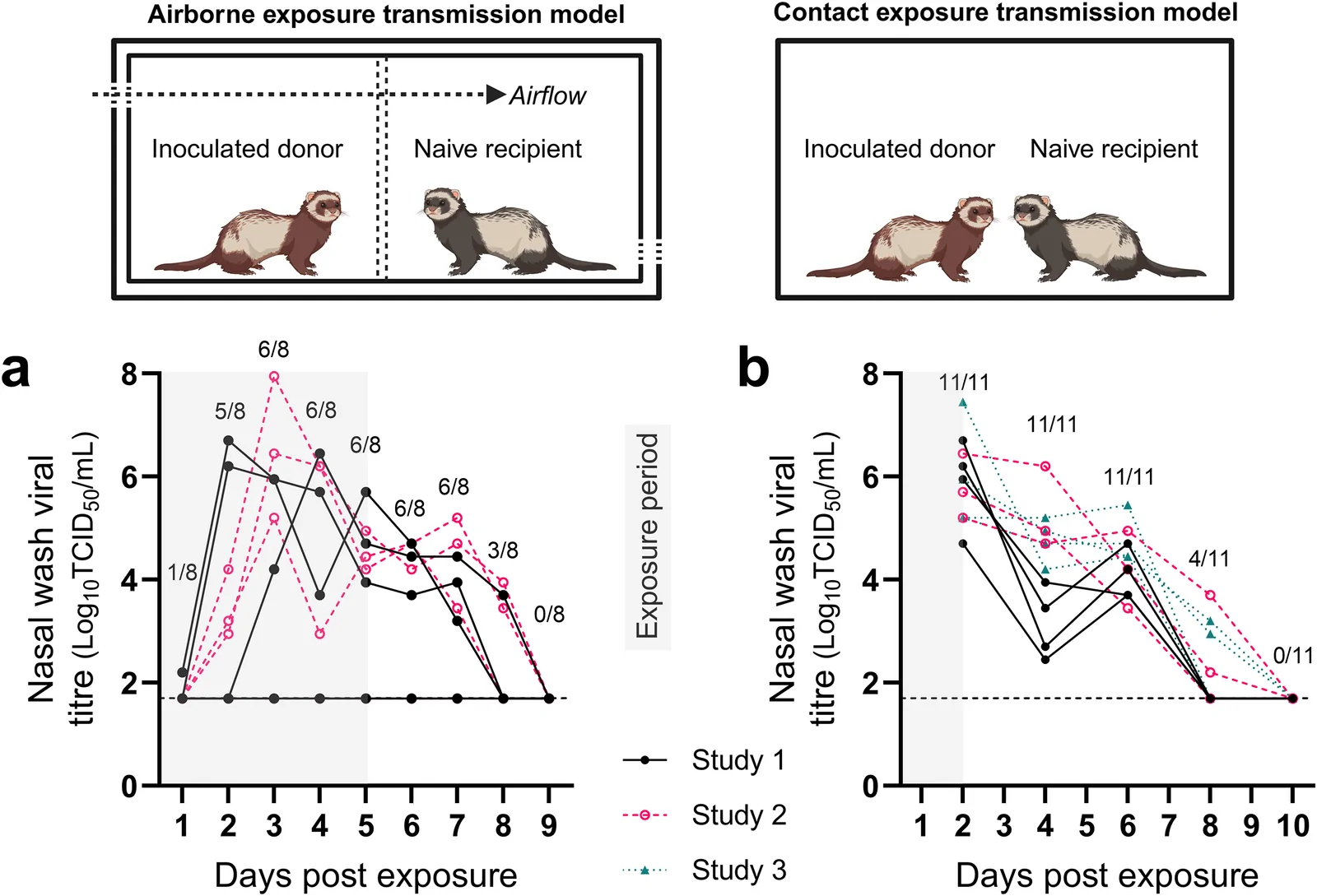
Airborne and contact transmission of influenza A(H1N1)pdm09 A/Sydney/5/2021 between ferrets. Donor ferrets were inoculated by the intranasal route with 5 log_10_TCID_50_/500µL with A/Sydney/5/2021. One day p.i. naïve recipient ferrets were exposed to infected donors (see S2 Table for viral shedding information from donor animals). (a) Airborne exposure recipient ferrets were housed in a neighbouring cage to an infected donor animal for five days (shaded region), separated by a permeable barrier, with mono-directional airflow from the donor to recipient ferret. Detection of viral titre in daily nasal washes from recipient animals at day one post exposure until day nine revealed transmission events, shown as a fraction of total recipient ferrets. (b) Contact exposure recipient ferrets were co-housed in the same cage as donors for two days (shaded region). Nasal wash titres from recipient ferrets, every second day from day two to ten post exposure, revealed all recipients shed detectable virus, shown as a fraction of total recipient ferrets. Airborne exposure studies include data from two independent studies (n = 8) and contact exposure studies from three independent studies (n = 11), with 3-4 ferrets per group, indicated by the different shapes, colours, and dashed lines. Created in BioRender (Stannard, H. (2026) https://BioRender.com/yqpszxw).

To further assess the utility of A/Sydney/5/2021 virus to serve not only as a challenge strain for assessments of viral pathogenicity but as a contemporary virus model for transmission studies in the ferret model, we assessed transmission rates between ferrets via both the airborne and contact exposure routes. Airborne transmission was assessed at a 1:1 donor:recipient ratio using eight pairs of ferrets, with naïve recipients separated from an infected donor by an aerosol permissive barrier, by measuring viral shedding in NWs and seroconversion of recipient ferrets. A/Sydney/5/2021 infected (donor) ferrets transmitted virus to six out of eight (75%) recipients (Fig 3a), and seven out of eight seroconverted (S2 Table). In a setting more conducive to transmission, combining donors and recipients within the same cage to encapsulate multiple transmission modes using eleven pairs of ferrets, 100% transmission of this virus occurred within two days to susceptible contact ferrets (Fig 3b). This indicated that A/Sydney/5/2021 virus was capable of efficient transmission to naïve recipients across multiple infection modes.

### Reduced URT viral loads in ferrets receiving oseltamivir following aerosol inhalation A(H1N1)pdm09 virus challenge compared to intranasal inoculation

**Fig 4:**
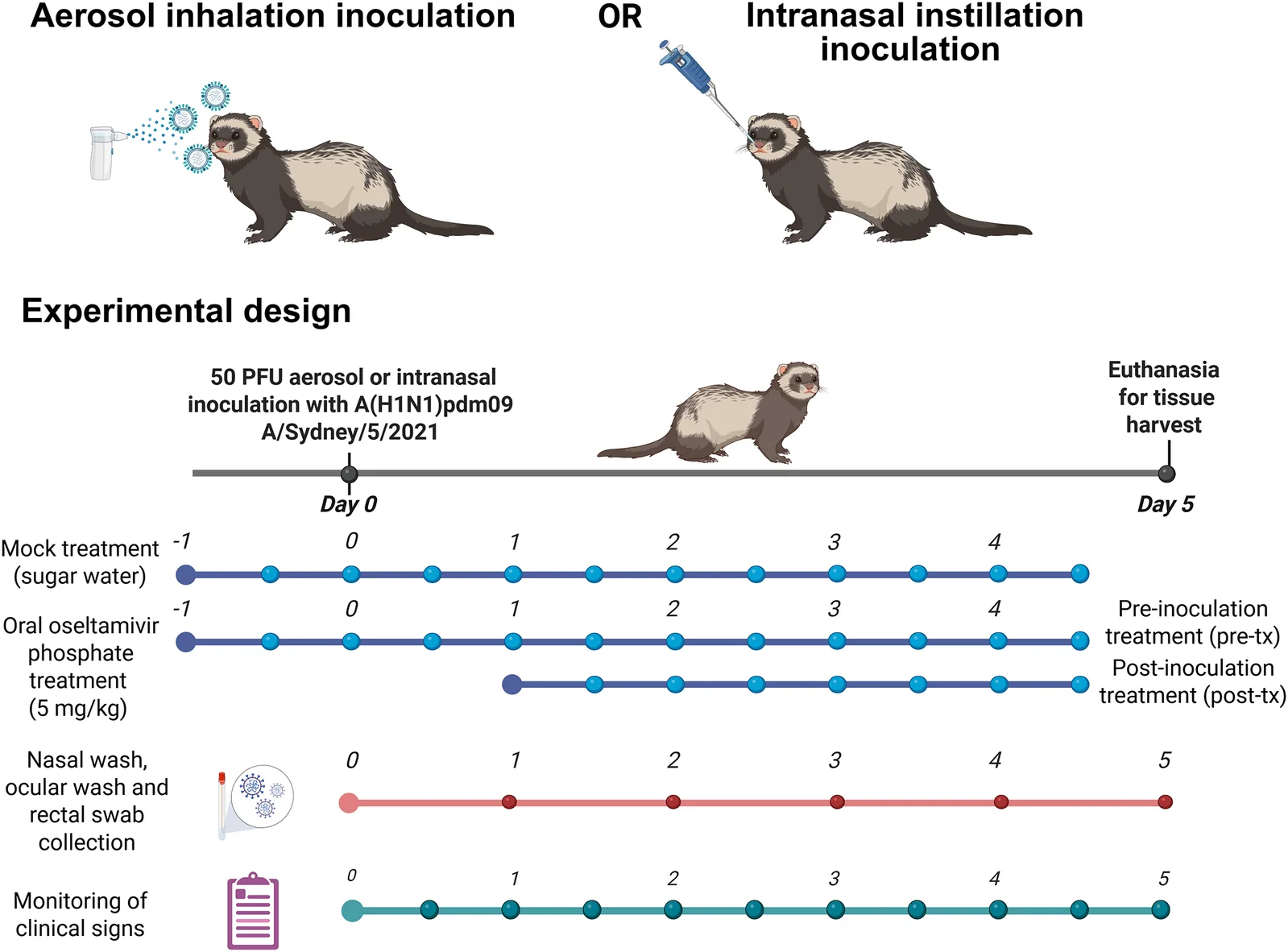
Comparative inoculation experimental design for evaluating oseltamivir efficacy against A(H1N1)pdm09. Ferrets were inoculated with A/Sydney/5/2021 virus by the intranasal route (50 PFU virus diluted in 1 ml total liquid volume deposited to the nares of animals) or by the aerosol route (15 min continuous exposure to aerosolized virus to achieve a presented dose of 50 PFU). OST/mock treatment was administered on the schedule shown (6am and 5pm dosing times), with indicated specimens and clinical signs reported on the schedule shown. Detailed information for the aerosol inoculation model setup is provided in the methods and in Gustin et al, 2011 [33]. Created in BioRender (Stannard, H. (2026) https://BioRender.com/f77iw02).

Aerosol inoculation of ferrets provides a more physiologically relevant exposure route than standard intranasal inoculation. As A/Sydney/5/2021 influenza A(H1N1)pdm09 virus can be transmitted to recipient ferrets following aerosol-only exposure to an infected donor animal, we sought to improve the reliability of this infection model and reduce ferret numbers by inoculating ferrets with a known presented dose of aerosolized virus generated under controlled conditions, permitting comparisons of this aerosol antiviral model to a standard intranasal inoculation model. Using a low titre inoculum of A/Sydney/5/2021, ferrets were inoculated by the aerosol inhalation route (15 min with an estimated presented dose of 50 PFU) or by intranasal inoculation (50 PFU/1ml) and observed daily through to day five p.i. For each inoculation method, ferrets received OST twice-daily (5mg/kg/dose), administered orally either pre-inoculation (pre-tx; Day -1 to Day 4 p.i., six days of twice daily treatment) or post-inoculation treatment (post-tx, Day 1 to Day 4 p.i., four days of twice daily treatment) (Fig 4); mock control ferrets received vehicle only on the same schedule as pre-tx ferrets.

**Fig 5:**
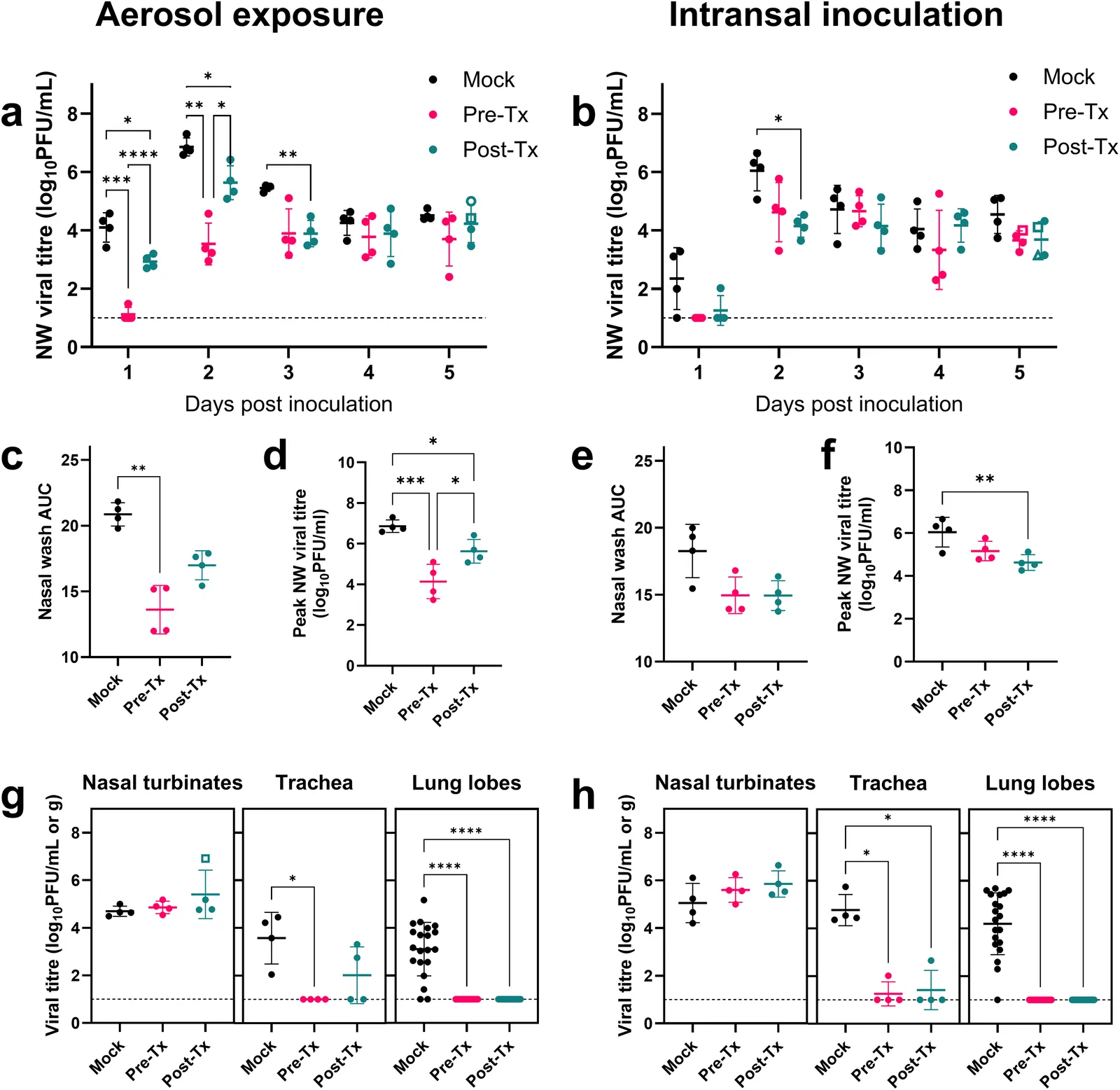
Assessing oseltamivir antiviral efficacy to reduce A(H1N1)pdm09 viral load in the respiratory tract of ferrets after aerosol exposure or intranasal inoculation. Ferrets were inoculated by the aerosol or intranasal (1 mL) route with A(H1N1)pdm09 A/Sydney/5/2021 virus at 50 PFU. Oseltamivir phosphate (5 mg/kg) or sugar water (mock: black) as a control was administered twice daily, either one day prior to inoculation until day four (pre-tx: pink), or one day post inoculation until day four (post-tx: green), n = 4 per group. Viral titres in nasal wash (NW) specimens were determined daily following either aerosol exposure (a) or intranasal inoculation (b) by standard plaque assay, with peak titers reported in d and f, respectively. Area under the curve (AUC) analysis was performed to compare the total viral shedding in NW specimens between ferret groups following either aerosol exposure (c) or intranasal inoculation (e). Viral load present in respiratory tissues (includes all five major lung lobes) from (g) aerosol exposure and (h) intranasal inoculation ferrets at day five post inoculation are shown individually. Limit of detection was 10 PFU/mL or g. Mean and SD, compared across each group by (a,b) two-way ANOVA Tukey post-hoc test, (d,f) ordinary one-way ANOVA Tukey post-hoc test, or (c,e,g,h) Kruskal-Wallis Dunn’s test (* p < 0.05, ** p < 0.01, *** p < 0.001, **** p < 0.0001). Open symbols indicate the detection of NA-H275Y amino acid change at various frequencies in NW or nasal turbinates at day 5 (□ - 6-20%, Δ - 21-35%, ○ - 36-50%).

We first assessed if inoculation route modulated the kinetics and magnitude of viral replication in the URT of ferrets receiving OST. In ferrets inoculated by the aerosol inhalation route, viral load in ferret NW specimens collected early (days 1–3) p.i. was significantly reduced in both OST treatment groups compared to untreated ferrets, with the greatest reduction in viral load observed in the pre-tx OST group (Fig 5a). Viral titres in NW specimens later in infection (days 4–5 p.i.) were equivalent across all groups, as were nasal turbinate titres collected day five p.i. AUC analysis clearly showed a significant reduction in overall viral shedding for the pre-tx group compared to mock (Fig 5c). Furthermore, mean peak NW titres were significantly reduced in both pre-tx (4.14 log_10_PFU/mL) and post-tx (5.63 log_10_PFU/mL) groups compared to mock (6.86 log_10_PFU/mL), with pre-tx also significantly reduced relative to post-tx (Fig 5d).

In contrast to aerosol inoculation, NW specimens collected from ferrets inoculated by the traditional intranasal route did not exhibit consistent differences in animals receiving OST relative to mock. While mean viral titres in ferrets receiving OST were lower compared to mock-treated ferrets at day two p.i. (mock 6.05 log_10_PFU/mL, pre-tx 4.63 log_10_PFU/mL, and post-tx 4.15 log_10_PFU/mL), statistical significance between groups was not consistently detected, and viral titres were comparable between all groups at all other timepoints assessed, including in nasal turbinates at day five p.i. (Fig 5b). AUC analysis showed pre- and post- treatments reduced overall shedding in NW specimens, although was not statistically significant (Fig 5e). NW mean peak titres showed a reduction in viral shedding following post-tx (4.63 log_10_PFU/mL), but not following pre-tx (5.17 log_10_PFU/mL) compared to mock (6.05 log_10_PFU/mL) (Fig 5f). NW data highlights the refinement possible in the aerosol exposure ferret antiviral model, in contrast to intranasal inoculation, where differences between treatment groups were less likely to be statistically significant. Additionally, comparable findings were also present in viral RNA copy number analysis day two p.i., and day five p.i. nasal wash samples, with each group across both inoculation routes maintaining an RNA-PFU ratio difference of log_10_ 3.56-4.78 (S4 Fig).

To determine how NA changes during NA inhibitor treatment, NW wash samples at day five p.i. were sequenced from all groups, revealing the emergence of NA-H275Y OST resistance mutations in two of four aerosol exposure post-tx ferrets, as well as two post-tx and one pre-tx ferrets following intranasal inoculation (at a variant frequency of 9–41%; Fig 5 and S3 Table). This evidence may suggest pre-tx regimes may reduce the frequency of OST resistance emerging compared to post-tx. The low frequency of detection may suggest that these NA-H275Y mutations arose late in infection and likely had minimal impact on reported viral loads in tested animals. An NA-V234L substitution also arose at small proportions in a couple of samples from untreated ferrets; therefore this was not due to drug selection pressure.

### Comparable LRT, ocular, and gastrointestinal tissue A(H1N1)pdm09 viral loads in ferrets receiving oseltamivir following either aerosol inhalation or intranasal inoculation

We next assessed if inoculation route modulated the kinetics and magnitude of viral replication in the lower respiratory tract of ferrets receiving OST (Fig 5g-h). No infectious virus was detected in lung tissues in ferrets receiving OST either pre-tx or post-tx independent of the inoculation route employed, supporting the high efficacy of OST in reducing virus replication in this tissue at day five p.i. relative to mock-treated ferrets. In agreement, viral load in trachea tissues was low to absent in ferrets receiving OST relative to mock at this time point irrespective of inoculation route.

Beyond the respiratory tract, we investigated if A/Sydney/5/2021 was capable of replication in ocular and gastrointestinal tissues, and if OST treatment modulated viral load at these sites. Between days 2–5 p.i., infectious virus was recovered from 10/16 and 11/16 conjunctival washes collected daily from mock-treated ferrets inoculated by the aerosol or intranasal route, respectively, reaching peak mean titres of 3.0 ± 0.3 and 3.1 ± 1.4 log_10_ PFU/mL. OST treatment reduced the incidence (1/32 and 2/32 conjunctival specimens with infectious virus among ferrets inoculated by the aerosol or intranasal route, respectively) and magnitude (peak titre among positive specimens 1.0-1.3 log_10_ PFU/mL) relative to mock-treated ferrets. In agreement with conjunctival wash specimens, viral titres in the eye or conjunctiva tissues day five p.i. were significantly reduced in OST treatment groups compared to mock (mean titres of 2.6 ± 0.8 and 3.2 ± 1.3 log_10_ PFU/mL for eye and 2.8 ± 0.9 and 4.4 ± 0.8 log_10_ PFU/mL for conjunctiva tissue in ferrets inoculated by the aerosol or intranasal route, respectively) (S5a-d Fig). Rectal swabs and intestine tissue revealed minimal viral load in the gastrointestinal tract; only rectal swabs from one aerosol (day 2 only) and one intranasal (day 4 and 5 only) inoculated animals had detectable viral titres (S5e-h Fig). Collectively, these findings revealed viral load at day five p.i. in respiratory, ocular and gastrointestinal tissues were comparable following both aerosol exposure and intranasal inoculation.

### Quantifiable clinical observations affirmed oseltamivir efficacy in A(H1N1)pdm09-infected ferrets after aerosol inhalation or intranasal inoculation

**Fig 6:**
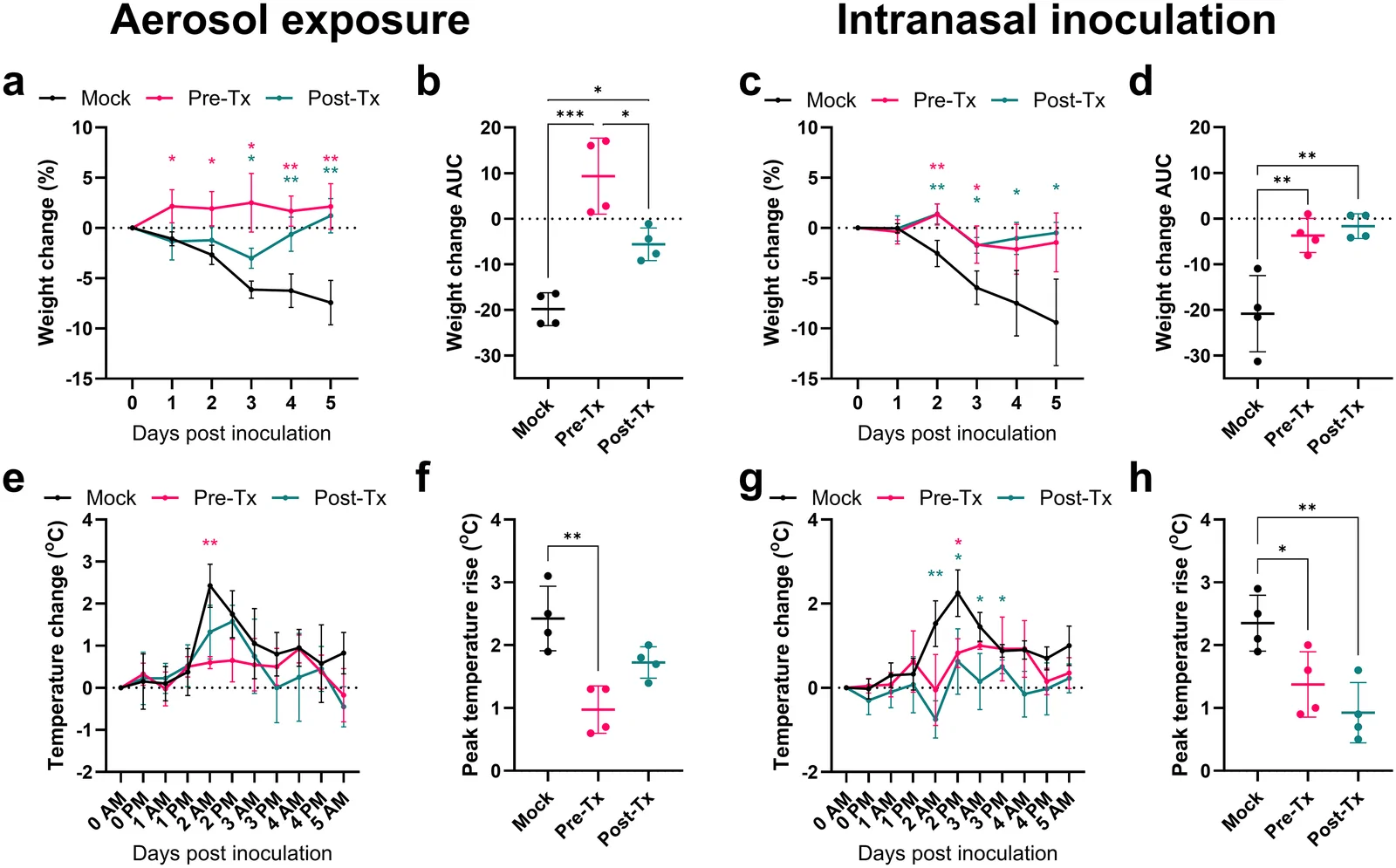
Weight loss and temperature changes in ferrets after aerosol exposure or intranasal inoculation with A(H1N1)pdm09 virus during oseltamivir antiviral treatment. Ferrets were inoculated by the aerosol or intranasal (1 mL) route with A(H1N1)pdm09 A/Sydney/5/2021 virus at 50 PFU. Oseltamivir phosphate (5 mg/kg) or sugar water as a mock control (black lines) was administered twice daily, either one day prior to inoculation until day four (Pre-Tx; pink lines), or one day post inoculation until day four (Post-Tx; green lines), n = 4 per group. Daily weight relative to baseline measurements are shown for aerosol (a) and intranasal (c) inoculated ferrets. Twice daily body temperature changes (degrees Celsius) for aerosol exposure (e,f) and intranasal inoculations (g,h) from a baseline of between 37 to 38.7 degrees Celsius. Area under the curve (AUC) analysis (b, d) or peak value comparison (f, h) was performed to compare the net weight or temperature change over time between ferret groups, respectively. Mean and SD shown in each graph, with weight and temperature compared by two-way ANOVA Tukey post-hoc test, and AUC and peak analysis compared across each group by ordinary one-way ANOVA Tukey post-hoc test (* p < 0.05, ** p < 0.01, *** p < 0.001). Temperatures were collected in the morning (AM) or evening (PM).

Analysis of ferret clinical signs revealed similar outcomes to virological findings in tissues, with protective benefits from both OST treatment regimes. Weight loss was greater in untreated animals regardless of inoculation route, particularly at day five p.i. (Fig 6a,c). Ferrets inoculated by the aerosol inhalation route showed statistically significant differences between pre- and post-tx weight change AUC, with pre-tx reducing weight loss, while intranasal inoculation did not distinguish between the two treatment groups, although both were protective (Fig 6b,d). Temperature analysis was more variable between ferrets. Ferrets inoculated by the aerosol inhalation route showed a reduction in temperature increase in the pre-tx group at day two (AM). Mean peak temperature readings were lower in both pre-tx and post-tx groups compared to mock, however the post-tx group did not reach statistical significance (Fig 6e-f). Similar trends in fever reduction were observed in intranasally inoculated animals receiving OST compared to mock (Fig 6g-h).

## Discussion

Continuous genetic drift of circulating influenza viruses necessitates periodic evaluation of contemporary virus isolates in relevant mammalian species to ensure pre-clinical models that assess prospective influenza drugs are as relevant to human infection scenarios as possible. The A(H1N1)pdm09 virus ferret infection model [5,6] represents the current gold-standard small animal *in vivo* system to aid the development of novel influenza drugs, but has relied primarily on a 2009-origin challenge strain (particularly, A/California/07/2009) to which currently circulating influenza isolates have drifted. Herein, we have extended our understanding of the A(H1N1)pdm09 influenza virus ferret model by evaluating a panel of human A(H1N1)pdm09 isolates from 2009 to 2022 for their viral replication, pathogenicity and transmissibility. Using a contemporary isolate from 2021 from HA clade 5a.2a, we demonstrated the suitability of this strain for pre-clinical antiviral drug studies against seasonal influenza A(H1N1)pdm09 viruses. This updated challenge virus was assessed via conventional intranasal inoculation, but also side-by-side with aerosol inhalation virus exposure, that more closely mimics influenza infections in humans. These two challenge methods compared OST antiviral treatment efficacy to propose a refinement to this model for subsequent evaluation of novel therapeutic agents.

Strain-specific differences in pathogenicity among A(H1N1)pdm09 viruses isolated since 2009 have been reported previously; two studies in ferrets [23,24], and three in mice [22,24,25] all revealed generally reduced pathogenicity of 2015 to 2019 isolates compared to 2009 isolates. These findings match our results in ferrets, which showed that A/Michigan/45/2015 and A/Victoria/2570/2019 inoculations resulted in the lowest lung viral loads and weight losses of the viruses tested. However, to our knowledge, the relative pathogenicity of A(H1N1)pdm09 virus isolates from 2021 and beyond have not yet been assessed in the ferret model.

While all viruses tested exhibited productive, high-titre replication in the ferret URT throughout the acute phase of infection, A/Sydney/5/2021 virus exhibited the highest capacity for high-titre replication and histopathological alterations in the lungs of ferrets (Fig 1-2). A/Sydney/5/2021 has accumulated 32 amino acid (AA) differences over time in the HA compared to A/California/07/2009, some of which may explain virus phenotypic differences observed in ferrets, such as greater nasal wash AUC, higher weight loss AUC, and more severe lung histopathology scores. A/Victoria/2570/2019 and A/Victoria/4897/2022 are the most similar in their HA to A/Sydney/5/2021 (8 and 9 AA differences respectively, S1 Fig), however while A/Victoria/4897/2022 had comparable weight loss, viral shedding, lung titres, and lung histopathology scores to A/Sydney/5/2021, A/Victoria/2570/2019 did not. We speculate that a phenotypic shift may have occurred following HA genetic changes from A/Victoria/2570/2019 to A/Sydney/5/2021. Changes outside the HA gene may also be responsible for observed differences between the five viruses tested, as shown in previous animal pathogenicity studies [49–54]. Further research is needed to identify specific genetic changes that may contribute to viral kinetics and pathogenicity differences in the ferret model.

Considering the capacity for A/Sydney/5/2021 virus to exhibit greater mean peak weight loss and peak infectious titre in the lungs compared to the commonly utilised A/California/07/2009 ferret challenge strain, A/Sydney/5/2021 was selected for subsequent evaluation for suitability as an updated A(H1N1)pdm09 influenza virus ferret model challenge strain. Importantly, the efficient transmissibility of A/Sydney/5/2021 between ferrets by both airborne and contact exposure (Fig 3) supports the use of this strain in pre-clinical assessments of influenza interventions on transmission between ferrets [16,34–37,39]. It should be noted that while some A(H1N1)pdm09-derived strains have exhibited a capacity to spread to and replicate in gastrointestinal tissue [55], infectious A/Sydney/5/2021 virus was not routinely detected in either serially collected rectal swab specimens days one-five p.i. or in intestinal tissue day five p.i. in mock-treated ferrets. As such, while we show the capacity for OST to reduce viral titres in ocular specimens following viral challenge in this study using either inoculation route tested, other contemporary strains warrant evaluation for potential use to assess antiviral-mediated mitigation of extrapulmonary spread to gastrointestinal tissues.

We utilised an established aerosol inoculation technique [33] to demonstrate potential benefits to the traditional intranasal inoculation method in the presence of OST treatment. Gustin et al. (2011) detailed the benefits of a low presented dose (4 or 44 PFU inhaled) of A(H3N2) A/Panama/2007/1999 with an aerosolised virus whole body exposure ferret model, which closely matched the virus shedding dynamics of a recipient ferret following airborne transmission, while the highest presented dose tested (190–431 PFU inhaled) reduced peak viral shedding compared to the low presented doses, and was similar to the 1mL intranasal inoculation route [33]. These findings suggest presented doses ≤50 PFU may more closely emulate the more biologically relevant exposure seen in ferret airborne transmission studies than higher presented doses. Additionally, low dose may be more biologically relevant to human infections, as a historical human challenge model demonstrated productive and symptomatic infections in three out of nine humans following inoculation with an estimated aerosolised infectious dose of just 5 TCID_50_ [56]. Therefore, we chose 50 PFU, as a biologically relevant and reproducible dose.

Ferreri et al. (2025), is the only other study to explore intranasal versus aerosol delivery of an influenza A(H1N1)pdm09 virus in ferrets (A/California/07/2009), which matched our results, revealing similar URT and LRT viral load at a late timepoint following acute infection (day four for their study and five p.i. here) between the two inoculation routes. However, likely due to use of an intranasal inoculation volume of 0.1 mL that restricted inoculum deposition to the URT, in this comparative study Ferreri et al. showed aerosol inhalation (which permits inoculum deposition to both the URT and LRT) resulted in elevated detection of infectious virus in the lungs at days 1–2 p.i. compared to intranasal inoculation. Our study did not assess viral loads in respiratory tract tissues at timepoints prior to day five p.i., which would be of interest in future studies. Of note, this study also utilised a high dose inoculum (10^6^ TCID_50_) that would facilitate robust replication in the LRT early post-inoculation. Our use of a 1 mL intranasal volume was chosen to deposit virus inoculum throughout the respiratory tract, including the lungs, as a like-for-like comparison to aerosol exposure [47]. Collectively, this work supports a need for continued investigation of how varying initial inoculation (including but not limited to route, volume, and viral dose) can modulate post-infection viral kinetics, magnitude, dispersal, and clinical outcomes, both in the context of viral pathogenicity and antiviral efficacy situations.

A/Sydney/5/2021 virus was sensitive to OST with a mean IC_50_ of approximately 1.0 ± 0.64 nM by NAI assay (S4 Table). In the ferret model, prophylactic treatment with OST (5 mg/kg twice daily) prior to infection with A/Sydney/5/2021 resulted in measurable reductions in morbidity and viral shedding when challenged by either intranasal or aerosol inhalation routes. Even at the low, human equivalent [57] dose administered, therapeutic administration of OST post infection resulted in statistically significant reductions in both morbidity and viral titre metrics (Fig 5-6). These levels of OST efficacy observed were comparable or better to other studies evaluating OST following A(H1N1)pdm09 challenge in ferrets [14,18,35,43,58]. The OST ferret studies with A/Sydney/5/2021 aerosol inhalation challenge revealed greater reductions in viral shedding for pre-tx than post-tx relative to mock, but this was not observed in intranasally inoculated ferrets, highlighting the granularity possible with the more natural aerosol infection route. Nonetheless, we found that low dose intranasal challenge demonstrated OST antiviral protection in clinical signs and tissues titres relative to untreated ferrets, highlighting the utility of low dose challenges when evaluating antiviral efficacy. The treatment reduction or mitigation of viral load in ferret tissues across both inoculation routes matches outcomes observed in previous intranasal or ocular influenza virus delivery ferret studies with OST [17,43,48,58], supporting the reproducibility of these findings regardless of inoculation dose or route, and the importance of measuring viral load in pulmonary and extra-pulmonary tissues, not simply nasal wash titres, when assessing antiviral efficacy.

Of note, both inoculation routes demonstrated the advantage of OST pre-tx compared to post-tx at reducing the incidence of OST resistance mutation (NA-H275Y) emerging in nasal wash samples. This has been observed previously in ferrets treated with OST at 36 h.p.i. [34], in line with the modelling predictions of some groups [59], although others proposed increase risk of antiviral resistance emerging following OST prophylaxis [60].

Studies supporting this work were conducted across two independent institutions, to demonstrate the suitability of the A/Sydney/5/2021 challenge strain to support public health efforts independent of laboratory-specific or protocol-specific confounding factors [35,39]. Nonetheless, this approach includes inherent limitations. Comparing the replicative fitness of different influenza virus strains in ferrets is difficult to quantify due to variability in host replication kinetics of viruses, given differences in virus stock generation, the use of outbred animals, and evaluation of individual strains across studies conducted at different times. To ensure consistency in contemporary virus pathotyping studies, we used identically prepared plaque purified viral stocks and infected ferrets with a high titre inoculum. A/Sydney/5/2021 inoculations were also repeated across two independent animal experiments, which demonstrated consistent results. Aerosol studies were only conducted at one institution, though the delayed augmentation of viral titres in NW specimens reported here with this inoculation method are in agreement with other previously published studies utilizing this approach [33,61]. Infectious virus titres were assessed in tissues at day five p.i. only; it is possible that active virus replication was taking place in the lower respiratory tract among OST-treated ferrets at earlier timepoints.

In conclusion, we have identified a contemporary viral strain (A/Sydney/5/2021) that generated consistent levels of replication, pathogenesis, and transmission in ferrets, which may improve the relevance and reliability of future studies aiming to explore anti-influenza interventions that aim to reduce viral burden and/or transmissibility of seasonal influenza A(H1N1)pdm09 viruses. We have also illustrated the benefit of a low dose aerosol exposure antiviral ferret model for assessing therapeutics following a more biologically relevant challenge. Continued investment in pre-clinical model challenge platforms that more closely emulate the diverse ways in which humans are exposed to influenza will improve our ability to rigorously assess novel therapeutic agents as well as investigating the efficacy of licensed therapeutics against novel and emerging influenza viruses.

## Methods

### Ethics statement

All animal procedures conducted in this study were approved by either the University of Melbourne Animal Ethics Committee (project license no. 20033) in accordance with the Australian Government, National Health and Medical Research Council Australian code of practice for the care and use of animals for scientific purposes (8th edition), or the Centers for Disease Control and Prevention Institutional Animal Care and Use Committee (IACUC) in an AAALAC International-accredited facility.

### Cells

Madin-Darby Canine Kidney (MDCK CCL-34) cells (ATCC, USA) were cultured at 37 °C and 5% CO_2_ in Dulbecco’s Modified Eagle Medium (DMEM, high glucose pyruvate; Gibco, USA). DMEM was supplemented with 10% foetal bovine serum (FBS, Bovogen Biologicals, Australia or Hyclone, USA), 1x GlutaMAX (Gibco, USA), 1x MEM non-essential amino acid solution (Gibco, USA), 0.05% sodium bicarbonate (Gibco, USA), 20 μM HEPES (Gibco), and 100 U/mL penicillin-streptomycin solution (Gibco, USA). Maintenance media (DMEM media containing the above constituents excluding only serum) was used for virus dilutions. Infection media (maintenance media supplemented with 2 µg/mL TPCK-treated trypsin (SAFC Biosciences, USA)) was used for virus infection protocols. The same stock of MDCK cells was used for viral titration at both institutions.

### Viruses

Five influenza A(H1N1)pdm09 virus strains, A/California/07/2009 (EPI_ISL_31553), A/Michigan/45/2015 (EPI_ISL_200780), A/Victoria/2570/2019 (EPI_ISL_417210), A/Sydney/5/2021 (EPI_ISL_12109632), and A/Victoria/4897/2022 (EPI_ISL_17102775) were plaque purified, as previously outlined [62], with virus whole genome sequences matching GISAID.org corresponding accession ID sequences. MDCK cells, washed twice with PBS, were used to generate virus stocks in DMEM infection media. All virus aliquots were stored at -80°C. The infectious virus titre was determined prior to use by virus titration assay (described below). The same stock of A/Sydney/5/2021 was used for ferret experiments at both institutions.

### Influenza HA phylogenetic tree

Sequences were aligned using MAFFT v7.526 [63]. The HA tree was inferred using the maximum likelihood method as implemented in IQ-TREE2 v2.3.3 [64] and least-square dating method [65]. Trees were visualised using the ggtree v3.10.1 package [66] in R v4.3.3. Clade assignment was performed using Nextclade v3.18.1 [67].

### Virus titration assays (Melbourne, Peter Doherty Institute for Infection and Immunity (PDI))

Infectious virus titres were determined by 50% tissue culture infective dose (TCID_50_) assay in MDCK cells, as previously described [44]. In brief, MDCK cells were seeded at 3.5 x 10^4^ cells/100 µL into a 96-well plate and cultured overnight at 37 °C in 5% CO_2_. The infectivity was determined by recording the presence of cyto-pathic effect (CPE) at four days post-infection following inoculation of MDCK cells in 96-well tissue culture plates with 20 µL of influenza virus, nasal wash (NW), or tissue homogenate, serially 10-fold diluted and tested in quadruplicate. The dilution at which 50% of the wells are infected is calculated using the Reed and Muench method [68].

### Virus titration assays (Atlanta, Centers for Disease Control and Prevention (CDC))

MDCK cells were seeded overnight into 6-well plates in DMEM (prepared as outlined above). Confluent monolayers were washed and infected with serial 10-fold dilutions of virus in 100 µL PBS. After 1 hour of incubation, the cells were washed, and an overlay consisting of a mixture of 1.6% agarose and 2x MEM medium (Gibco) with TPCK-treated trypsin (Sigma-Aldrich, St. Louis, MO) was added to the cells. After 72 h of incubation at 37°C, the overlay was removed, and plaques were stained with 0.1% crystal violet and counted to calculate the plaque forming units (PFU)/mL.

To compare titration assays at both institutions, the same A/Sydney/5/2021 virus stock was titrated to determine the TCID_50_/mL to PFU/mL equivalent virus titre. The mean titre (n = 3) of this virus stock was 3.3 x 10^7^ PFU/mL (Atlanta, CDC) and 6.9 x 10^6^ TCID_50_/mL (Melbourne, PDI), resulting in a 4.8-fold change greater titre following PFU titration than the TCID_50_ assay.

### Ferrets (Melbourne, PDI)

Outbred male and female ferrets (*Mustela putorius furo*) were obtained from commercial breeders (Animalactic Animals & Animal Products Pty Ltd, Australia) and were a minimum of 12 weeks of age and 0.6 kg in body weight. Seronegativity against the three different types/subtypes of recently circulating human influenza virus strains was confirmed by haemagglutination inhibition assay. Ferrets were housed individually in high efficiency particulate air filtered cages with ad libitum food, water and enrichment equipment throughout the experimental period. For all studies ferrets were monitored daily for clinical signs, body weight, and body temperature, using a subcutaneous temperature transponder (Digivet, Australia) inserted into the dorsal space between the scapulae prior to experimental use.

### Ferrets (Atlanta, CDC)

Male Fitch ferrets (Triple F Farms, Sayre, PA) were 12 months of age at time of use (minimum body weight 1.1 kg). Ferrets were serologically negative to currently circulating influenza A and B viruses as determined by standard hemagglutination inhibition assay. All animals were housed in Duo-Flo Bioclean mobile units (Lab Products Incorporated, Seaford, DE) throughout each experiment, on a 12:12h light:dark cycle. Ferrets were anesthetized for all procedures with an intramuscular injection of a ketamine cocktail (25 mg/kg ketamine, 2 mg/kg xylazine). To monitor body temperature, a subcutaneous temperature transponder (IPTT-300, BMDS, Seaford, DE) was inserted into the dorsal space between the scapulae prior to experimental use.

### Ferret virus replication and pathogenesis study (Melbourne, PDI)

All ferrets received anaesthesia (1:1 (v/v) ketamine (100 mg/mL) and xylazine (20 mg/mL)) via intramuscular injection. 5 log_10_TCID_50_ units of each influenza A(H1N1)pdm09 virus in 500 µL (diluted in PBS) was delivered by the intranasal route (250 µL per nostril). Additionally, influenza A(H3N2) virus, A/Darwin/6/2021, was used as a negative control as this isolate did not demonstrate viral replication in the LRT of ferrets.

Ferrets were sedated daily (Xylazine; 5 mg/kg), monitored for clinical signs, and NW samples were collected with 1 mL of PBS. At day three and five post-inoculation all animals were anaesthetized and humanely euthanized (Lethabarb; 0.5mL/kg) by intrahepatic injection. Three lung lobes (left cranial, right middle and caudal) and nasal turbinate tissue were excised, weighed, and separately homogenised in PBS (10% w/v dilution) using an Omni Soft Tissue Tip Homogenizing Kit (PerkinElmer, USA). Residual cells and connective tissue were removed by twice centrifugation at 4000 rpm for 10 mins. NW and tissue homogenate supernatants were stored at -80^o^C. The infectious viral load of all samples was determined by TCID_50_ virus titration assay after only one freeze-thaw.

### Ferret lung histology sample preparation and analysis (Melbourne, PDI)

Post ferret euthanasia, intact trachea and lungs were removed immediately, and bronchi branches connecting to the left cranial, right middle and caudal were tied closed with surgical silk. These three lung lobes were excised for titration. The remaining right cranial and left caudal lobes were inflated with 10–15 mL 10% neutral buffered formalin (NBF; Sigma) syringed via trachea, suspended in 100 mL 10% NBF for 2–3 days, and stored in 70% ethanol. Sectioned lung lobes were paraffin embedded, sliced and mounted on slides stained by hematoxylin and eosin (H&E). A veterinary pathologist scored the entire lung section for bronchiolar and alveolar inflammation from zero to three, under four different categories, as outlined in Gauger et al. (2012) [69].

Immunohistochemistry for influenza virus nucleoprotein (NP) and H&E staining was performed on matched sections of ferret lung lobes. Sections were first deparaffinized and rehydrated with xylene (Sigma) and ethanol treatments. Antigen retrieval was performed was performed using sodium citrate buffer (10 mM sodium citrate, 0.05% Tween 20, pH 6.0) heated to 96^o^C for 30 mins. Following Triton-X-100 (Thermo) permeabilization, sections were stained with laboratory-made anti-NP rabbit polyclonal sera [70] (A2915, Prof. Paul Digard, University of Edinburgh), and anti-rabbit goat horseradish peroxidase-conjugated secondary antibody. Brown signal (NP positive cells) was detected by staining with Rapid DAB detection kit (Abcam), with a hematoxylin counterstain. Sections were mounted using ProLong Gold antifade reagent (Invitrogen) and scanned using a Zeiss Axioscan 7 using bright-field settings.

### Ferret transmission studies (Melbourne, PDI)

As described above, ‘donor’ ferrets were inoculated with 5 log_10_TCID_50_ units of influenza A(H1N1)pdm09 A/Sydney/5/2021 virus in 500 µL PBS. In all studies naïve ‘recipient’ ferrets were introduced day one p.i. To assess airborne transmission, the recipient ferret was placed into a donor-adjacent cage for five days, separated by a double panel perforated metal sheet (two staggered panels 25.4 mm apart with holes of 5 mm in diameter and spaced 3 mm apart) and one-directional circular airflow with 25–40 air changes per hour in a 61.3 cm x 50.1 cm x 88.6 cm (27.2 m^3^) cage (Allentown Inc. NJ, USA); this cage design does not distinguish between airborne droplet (> 5 µm particle diameter) and aerosol (<5 µm) virus transmission [71]. Two independent airborne transmission studies were performed using identical experimental setups (n = 4 donor ferrets per group for a total of n = 8 transmission pairs). To assess direct contact transmission, the recipient ferret was placed into the same cage as the donor ferret for two days. Three independent direct contact transmission studies were performed using identical experimental setups (n = 3–4 ferrets per group for a total of n = 11 transmission pairs). Nasal washing was performed daily on all ferrets (airborne transmission) or every second day (direct contact transmission) until day ten post-exposure; the infectious viral load of all NW specimens was determined by TCID_50_ virus titration assay after only one freeze-thaw. Donor ferrets were euthanised at seven days post inoculation, and recipient animals were euthanized at 16 days (airborne transmission) or 14 days (direct contact transmission) post-exposure. Airborne transmission recipient ferrets were bled immediately prior to euthanasia for serological analysis against the exposed virus by hemagglutination inhibition assay using 1% turkey red blood cells (diluted in PBS).

### Aerosol inoculation of ferrets (Atlanta, CDC)

All aerosol experiments were conducted using the AeroMP aerosol management platform (Biaera Technologies, Hagerstown, MD), housed inside a Class II biological safety cabinet. Determination of virus spray factor (SF) for the A/Sydney/5/2021 virus stock (diluted in a PBS-0.3% BSA solution) was conducted as previously described [33,72], using a three-jet Collison nebulizer to generate aerosols and an impinger (Biosampler, SKC) as the post-aerosolization sampler. Anesthetized ferrets inside a disposable Tyvek sleeve (exposing the snout only) were placed in a stainless steel mesh restraint cage and moved to the aerosol exposure chamber (total n = 4 animals in the chamber during an aerosol exposure run). All aerosol experiments had a fixed 15-min exposure time (followed by a 5-min purge to allow aerosolized virus to evacuate the chamber) at a flow rate of 20 L/min, with continuous aerosol collection in the sampler at 12.5 L/min in a 5ml volume (Dulbecco modified Eagle medium supplemented with 0.3% BSA and 0.01% antifoaming agent Y-30 (Sigma)) and 7.5 L/min exhaust. All aerosol experiments were conducted at 20°C and 50% relative humidity. Respiratory inhalation presented doses were calculated as previously described by multiplying the concentration of virus in the aerosol by the respiratory minute volume (based on animal weight) and exposure time [33]; for these studies, the presented dose reported for each animal represents the quantity of virus inhaled by the ferret during 15-min exposure time, not the amount deposited onto the respiratory surface.

### Aerosol versus intranasal antiviral study (Atlanta, CDC)

Aerosol inoculation of anaesthetized ferrets was conducted as detailed above, with influenza A(H1N1)pdm09 virus A/Sydney/5/2021. The mean respiratory inhalation presented dose was calculated to be 50 PFU per animal (range, 39–69 PFU). The intranasal antiviral study began immediately after the dose was determined. Anaesthetized ferrets were infected by intranasal administration of 50 PFU of A/Sydney/5/2021 virus in 1 mL of PBS (500 µl/nostril).

Ferrets received oseltamivir phosphate (OST; MedChemExpress) dissolved in sterile sugar syrup (15% fructose in water) to a concentration of 5 mg/kg and administered by mouth in a syringe to alert ferrets (1 mL total volume/ferret). Ferrets received OST twice-daily (approx. 6am and 5pm) starting 24 hours prior to virus inoculation (pre-tx) or 24 hours post virus inoculation (post-tx). Mock treated ferrets received sterile sugar syrup only on the same schedule as pre-tx ferrets.

Post-inoculation, ferrets were observed twice-daily for clinical signs of infection (weight loss and temperature rise). NW specimens were obtained daily (approx. 8–9am) by introducing 1ml of PBS into the nasal passages to induce sneezing and collecting the aspirate using a sterile petri dish. Conjunctival wash (CW) and rectal swab (RS) specimens were collected as previously described [73]. Ferrets were euthanized for tissue harvest (nasal turbinates, trachea, whole lung lobes, intestine, eyes, and conjunctiva) at day five post infection after sedation via intracardiac administration of 1.0ml/kg, 390 mg pentobarbital sodium and 50 mg phenytoin sodium per 100ml (Euthanasia Solution, Med-Pharmex, Ponoma, CA). All specimens were immediately placed on dry ice upon collection and subsequently stored at -70°C until titration by plaque assay on MDCK cells. Specimens are reported as log_10_ PFU/mL (nasal wash, CW, RS, nasal turbinates, eye, conjunctiva) or/g (trachea, lung, intestine).

### Ferret specimen virus quantification and sequencing (Atlanta, CDC)

To extract viral RNA from ferret NW or tissues, 140 µl of ferret NW or tissue homogenate was inactivated with 560 µl AVL buffer following manufacturers protocol using the QIAamp Viral RNA Mini Kit (Qiagen, Venlo, Netherlands) on the QIAcube HT automated high-throughput nucleic acid purification platform (Qiagen, Venlo, Netherlands). For quantification of viral RNA copy numbers, real-time RT-PCR was performed with a SuperScript III Platinum One-Step qRT-PCR System (Invitrogen) in duplicate using an influenza A virus M1 gene primer and probe set with extrapolation of copy numbers based on a standard curve of samples with known M gene copy numbers. For sequencing, extracted RNA was reverse transcribed and genome amplified for all influenza gene segments using universal primers described previously [74]. Amplicons were processed for sequencing on the Illumina iSeq100 (Illumina, San Diego, California, USA) platform as previously described [61]. Data were processed in Geneious Prime v2023.0.4, trimming reads with BBduk v1 to remove primers, adapters, low-quality reads (Q < 30), or short reads (<40 bp). Trimmed reads were mapped to the reference/inoculum genome with 50 bp N spacers between gene segments using Bowtie2 v2.4.53 with end-to-end alignment on the highest sensitivity. Variants were called at 5% frequency using a minimum coverage of 100x and excluding paired reads mapped more than 30% from the expected distance of 500 bp. All sequence information has been deposited to the NCBI Sequence Read Archive (BioProject identification number PRJNA1481170).

### Neuraminidase inhibition assay

Briefly, each virus isolate virus was titrated to its optimum NA activity. 50 µL of diluted virus was incubated with 50 µL of oseltamivir carboxylate, peramivir, laninamivir or zanamivir at a range of concentrations (0.03–30,000 nM) for 45 min at room temperature. 50 µL of 300 µM MUNANA (2’-(4-Methylumbelliferyl)-α-D-N-acetylneuraminic acid) substrate was added to the virus and drug mixture and further incubated for 60 min at 37 °C. To terminate the reaction, 100 µL of 0.14 M NaOH in ethanol was added. The NA enzymatic activity at each NAI concentration was read using a fluorometer. JASPR v1.2 curve fitting software (kindly provided by Dr. Larisa Gubareva, CDC, USA) was used to determine the half-maximal inhibitory concentration (IC_50_) of each virus.

### Statistics and reproducibility

All statistical analysis performed throughout are named with the statistical test used with information about the exact sample size, any assumptions or corrections, and the resulting p value of the null-hypothesis tests (* p < 0.05, ** p < 0.01, *** p < 0.001, **** p < 0.0001), using GraphPad Prism v10. Comparisons over time were performed using two-way ANOVA followed by Tukey’s post-hoc multiple-comparison test, assuming normality. Comparisons of grouped or column data were analysed by using Kruskal-Wallis with Dunn’s multiple comparison test for data that failed normality test (Shapiro-Wilk), while data that met conditions for normality were compared by ordinary one-way ANOVA Tukey’s multiple comparison test. Standard deviation was used to capture error from the mean in graphical representations. *In vivo*, the virus replication and pathogenesis study, and antiviral comparative study, were performed with a minimum of four ferrets per group (more animals where indicated). Area under the curve (AUC) was calculated from line graphs with XY data from individual animals, summing the area above or below y-axis Y = 0, from the lowest X value (i.e., day 1) and the highest X value (i.e., day 5). Peak values from animal studies were the maximal value from each animal across the duration of the study.

## Supporting information

S1 Fig Influenza A(H1N1)pdm09 HA phylogenetic tree of vaccine candidate strains. Influenza A(H1N1)pdm09 viruses recommended for selection in northern or southern hemisphere annual influenza vaccines were chosen as representative strains. The HA tree was inferred using maximum likelihood method and least-square dating method, visualised using the ggtree package in R. Clade assignment was performed using Nextclade v3.18.1. Viruses evaluated in vivo are indicated with filled circles and bold font.
(DOCX)

S1 Table Influenza A(H1N1)pdm09 viruses whole genome amino acid alignment. (DOCX)

S2 Fig Weight loss and temperature of ferrets following intranasal inoculation of ferrets with different A(H1N1)pdm09 viruses. Ferrets were infected intranasally with 5 log_10_TCID_50_/mL units of A/California/07/2009, A/Michigan/45/2015, A/Victoria/2570/2019, A/Sydney/5/2021 or A/Victoria/4897/2022 virus in 500 µL. At day three or five post infection animals were culled for tissue harvest, n= 4 per group (except A/Sydney/5/21 n=8 at day five). (a) Weight and (b) body temperature changes from baseline were recorded daily. Area under the curve (AUC) analysis was performed to compare the net (c) weight change and (e) temperature change over time between ferret groups. Peak recorded (d) weight loss and (f) temperature rise over the duration of infection were also analysed. Mean and SD are shown for weight and temperature changes, analysis performed by One-way ANOVA Tukey post-hoc test (* p<0.05, ** p<0.01).
(DOCX)

S3 Fig Influenza A(H1N1)pdm09 virus induced histopathological changes in the lung lobes of ferrets. Ferrets infected with 5 log_10_TCID_50_/500µL of A/California/07/2009, A/Michigan/45/2015, A/Victoria/2570/2019, A/Sydney/5/2021, or A/Victoria/4897/2022 were culled at day five post intranasal inoculation. Intact trachea and lungs were removed, inflated with formalin for fixation. Sectioned lung lobes were embedded in paraffin, sliced at 0.5µM and mounted on slides. (a) Representative slides were stained by hematoxylin and eosin, or (b) immunohistochemistry (IHC) with influenza A virus nucleoprotein (IAV NP) antibody and a hematoxylin counterstain, and (c) higher magnification scans (taken from black squares) revealed sites of infection (black arrows). Two images of the same lung section are shown in the top and bottom panel.
(DOCX)

S2 Table Ferret influenza transmission studies, nasal wash virus shedding and seroconversion analysis. (DOCX)

S3 Table Virus amino acid variants of sequenced ferret nasal turbinates and nasal washes from A/Sydney/5/2021 oseltamivir antiviral efficacy study. (DOCX)

S4 Fig Comparison of infectious virus and RNA titres in nasal wash samples while assessing oseltamivir antiviral efficacy to reduce A(H1N1)pdm09 viral load in the respiratory tract of ferrets following aerosol exposure or intranasal inoculation. Ferrets were inoculated by the aerosol or intranasal (1 mL) route with A(H1N1)pdm09 A/Sydney/5/2021 virus at 50 PFU. Oseltamivir phosphate (5 mg/kg) or sugar water (mock: black) as a control was administered twice daily, either one day prior to inoculation until day four (pre-tx: pink), or one day post inoculation until day four (post-tx: green), n = 4 per group. Viral titres in nasal wash (NW) specimens were determined daily following either aerosol exposure or intranasal inoculation by standard plaque assay (Infectious titre) or quantitative PCR of the viral matrix gene (Viral RNA titre) on day two and five only. Mean and SD, compared across each group by two-way ANOVA Tukey post-hoc test (* p<0.05, ** p<0.01, *** p<0.001, **** p<0.0001). Open symbols indicate the detection of NA-H275Y amino acid change at various frequencies in NW or nasal turbinates at day 5 (□ - 6-20%, Δ - 21-35%, ○ - 36-50%).
(DOCX)

S5 Fig Viral load in ocular and gastrointestinal sites of ferrets following A(H1N1)pdm09 virus aerosol exposure or intranasal inoculation to evaluate oseltamivir antiviral efficacy. Ferrets were inoculated by the aerosol or intranasal route with A(H1N1)pdm09 A/Sydney/5/2021 virus at 50 PFU. Oseltamivir phosphate (5 mg/kg) or sugar water as a control was administered twice daily, either one day prior to infection to day three (Pre-tx), or one day post infection until day four (Post-tx), n = 4 per group. Viral titres in daily conjunctiva wash (CW) were determined following either (a) aerosol exposure or (c) intranasal inoculation by plaque assay. Additionally, daily rectal swabs (RS) were titrated following (e) aerosol and (g) intranasal infection. At day five post infection (b,d) conjunctiva and (f,h) intestine tissue was harvested for virus titration. Mean and SD are compared across each group. Tissue viral load was compared by Kruskal-Wallis with Dunn’s multiple comparison post-hoc test (* p<0.05, ** p<0.01).
(DOCX)

S4 Table Influenza A(H1N1)pdm09 A/Sydney/5/2021 neuraminidase inhibition (NAI) assay IC50 assessment. (DOCX)
