# Supplementary material for: Updated A(H1N1)pdm09 influenza virus ferret infection model permits refined antiviral assessment using aerosol inhalation challenge
Source: PLoS Pathog. 2026 Sep 9;22(9):e1014604. doi: 10.1371/journal.ppat.1014604 (PMC13588513; doi:10.1371/journal.ppat.1014604)
Supplement: Supplementary file 1 [file ppat.1014604.s001.docx]

**Supporting Figure 1. Influenza A(H1N1)pdm09 HA phylogenetic tree of vaccine candidate strains.** Influenza A(H1N1)pdm09 viruses recommended for selection in northern or southern hemisphere annual influenza vaccines were chosen as representative strains. The HA tree was inferred using maximum likelihood method and least-square dating method, visualised using the ggtree package in R. Clade assignment was performed using Nextclade v3.18.1. Viruses evaluated *in vivo* are indicated with filled circles and bold font.
