# Supplementary material for: Updated A(H1N1)pdm09 influenza virus ferret infection model permits refined antiviral assessment using aerosol inhalation challenge
Source: PLoS Pathog. 2026 Sep 9;22(9):e1014604. doi: 10.1371/journal.ppat.1014604 (PMC13588513; doi:10.1371/journal.ppat.1014604)
Supplement: Supplementary file 2 [file ppat.1014604.s002.docx]

**Supporting Table 1. Whole genome amino acid alignment of influenza A(H1N1)pdm09 viruses used in this study.**

| **PB2 Position** | **54** | **66** | **81** | **195** | **225** | **293** | **299** | **344** | **354** | **398** | **453** | **511** | **667** | **731** |  |  |
| --- | --- | --- | --- | --- | --- | --- | --- | --- | --- | --- | --- | --- | --- | --- | --- | --- |
| A/California/07/2009 | R | M | T | D | G | R | R | V | I | I | S | V | V | V |  |  |
| A/Michigan/45/2015 | K | I | T | N | G | K | R | M | L | T | P | V | V | I |  |  |
| A/Victoria/2570/2019 | K | I | M | N | S | K | K | M | L | I | T | V | I | I |  |  |
| A/Sydney/5/2021 | K | I | I | N | S | K | K | M | L | I | T | I | I | I |  |  |
| A/Victoria/4897/2022 | K | I | I | N | S | K | K | M | L | I | T | V | I | I |  |  |
| **PB1 Position** | **57** | **154** | **200** | **386** | **397** | **428** | **435** |  |  |  |  |  |  |  |  |  |
| A/California/07/2009 | T | G | V | K | I | K | I |  |  |  |  |  |  |  |  |  |
| A/Michigan/45/2015 | T | D | V | K | M | Q | T |  |  |  |  |  |  |  |  |  |
| A/Victoria/2570/2019 | T | D | I | R | M | Q | T |  |  |  |  |  |  |  |  |  |
| A/Sydney/5/2021 | T | D | I | R | M | Q | T |  |  |  |  |  |  |  |  |  |
| A/Victoria/4897/2022 | K | D | I | R | M | Q | T |  |  |  |  |  |  |  |  |  |
| **PA Position** | **18** | **30** | **61** | **63** | **100** | **224** | **225** | **226** | **321** | **330** | **337** | **362** | **395** | **438** | **505** | **626** |
| A/California/07/2009 | G | I | I | V | V | P | S | L | N | I | A | R | S | I | I | R |
| A/Michigan/45/2015 | E | I | I | V | I | S | S | L | K | V | A | K | S | I | I | R |
| A/Victoria/2570/2019 | E | I | M | L | I | S | C | L | K | V | A | K | G | I | V | R |
| A/Sydney/5/2021 | E | I | I | L | I | S | C | I | K | I | A | K | S | I | V | K |
| A/Victoria/4897/2022 | E | V | M | L | I | S | C | L | K | V | T | K | S | V | V | R |
| **HA1 Position (A)** | **54** | **74** | **83** | **84** | **94** | **97** | **129** | **130** | **137** | **142** | **156** | **161** | **162** | **163** | **164** | **183** |
| A/California/07/2009 | K | S | P | S | D | D | N | K | P | K | N | L | S | K | S | P |
| A/Michigan/45/2015 | K | S | S | N | D | N | N | K | P | K | N | L | N | Q | S | S |
| A/Victoria/2570/2019 | K | R | S | N | D | N | D | N | P | K | K | I | N | Q | T | P |
| A/Sydney/5/2021 | Q | R | S | N | N | N | D | N | P | K | K | I | N | Q | T | P |
| A/Victoria/4897/2022 | Q | R | S | N | D | N | D | N | S | R | K | I | N | Q | T | P |
| **HA1 Position (B)** | **185** | **186** | **189** | **203** | **216** | **223** | **224** | **250** | **256** | **259** | **260** | **277** | **283** | **295** | **308** | **321** |
| A/California/07/2009 | S | A | Q | S | I | Q | E | V | A | R | N | T | K | I | K | I |
| A/Michigan/45/2015 | T | A | Q | T | T | R | E | V | T | R | N | T | E | I | K | V |
| A/Victoria/2570/2019 | I | A | Q | T | T | R | E | A | T | R | D | T | E | V | K | V |
| A/Sydney/5/2021 | I | T | E | T | A | R | A | A | T | K | D | T | E | V | R | V |
| A/Victoria/4897/2022 | I | T | E | T | A | Q | A | A | T | K | D | A | E | V | R | V |
| **HA1 Position (C)** | **357** | **374** | **418** | **451** | **499** | **506** |  |  |  |  |  |  |  |  |  |  |
| A/California/07/2009 | E | E | I | S | E | E |  |  |  |  |  |  |  |  |  |  |
| A/Michigan/45/2015 | E | K | I | N | K | E |  |  |  |  |  |  |  |  |  |  |
| A/Victoria/2570/2019 | E | K | I | N | K | D |  |  |  |  |  |  |  |  |  |  |
| A/Sydney/5/2021 | E | K | I | N | K | D |  |  |  |  |  |  |  |  |  |  |
| A/Victoria/4897/2022 | D | K | V | H | K | D |  |  |  |  |  |  |  |  |  |  |
| **NP Position** | **22** | **100** | **102** | **105** | **425** | **453** | **498** |  |  |  |  |  |  |  |  |  |
| A/California/07/2009 | A | V | R | M | V | P | S |  |  |  |  |  |  |  |  |  |
| A/Michigan/45/2015 | T | I | G | T | V | P | N |  |  |  |  |  |  |  |  |  |
| A/Victoria/2570/2019 | T | I | G | T | I | P | N |  |  |  |  |  |  |  |  |  |
| A/Sydney/5/2021 | T | I | G | T | I | P | N |  |  |  |  |  |  |  |  |  |
| A/Victoria/4897/2022 | T | I | G | T | I | Q | N |  |  |  |  |  |  |  |  |  |
| **NA Position (A)** | **13** | **19** | **34** | **40** | **44** | **50** | **51** | **66** | **74** | **77** | **81** | **188** | **200** | **222** | **241** | **248** |
| A/California/07/2009 | V | M | I | L | N | N | Q | Y | F | G | V | I | N | N | V | N |
| A/Michigan/45/2015 | I | M | V | I | S | N | Q | Y | F | G | V | I | S | N | I | D |
| A/Victoria/2570/2019 | I | T | V | I | S | N | K | T | S | R | A | T | S | K | I | D |
| A/Sydney/5/2021 | I | T | V | I | S | N | K | T | S | R | A | T | S | K | I | D |
| A/Victoria/4897/2022 | I | T | V | I | S | D | K | T | S | R | A | T | S | K | I | D |
| **NA Position (B)** | **264** | **270** | **314** | **321** | **369** | **382** | **386** | **389** | **416** | **430** | **432** | **449** | **452** | **453** | **469** |  |
| A/California/07/2009 | V | N | I | I | N | G | N | I | D | R | K | N | T | V | K |  |
| A/Michigan/45/2015 | I | K | M | V | K | G | K | I | D | R | E | N | T | V | K |  |
| A/Victoria/2570/2019 | I | K | M | V | K | G | K | K | N | L | E | D | I | V | K |  |
| A/Sydney/5/2021 | I | K | M | V | K | G | K | K | N | R | E | D | I | M | K |  |
| A/Victoria/4897/2022 | I | K | M | V | K | E | K | K | N | R | E | D | I | M | N |  |
| **MP Position** | **42** | **80** | **98** | **192** | **208** | **230** | **235** | **261** | **280** | **309** |  |  |  |  |  |  |
| A/California/07/2009 | L | V | K | M | Q | K | E | Y | Y | T |  |  |  |  |  |  |
| A/Michigan/45/2015 | L | I | K | V | K | R | E | H | H | A |  |  |  |  |  |  |
| A/Victoria/2570/2019 | L | I | K | V | K | R | E | H | H | A |  |  |  |  |  |  |
| A/Sydney/5/2021 | L | I | K | V | K | R | A | H | H | A |  |  |  |  |  |  |
| A/Victoria/4897/2022 | I | I | R | V | K | R | E | H | H | A |  |  |  |  |  |  |
| **NS Position** | **2** | **18** | **55** | **65** | **80** | **84** | **90** | **95** | **123** | **125** | **131** | **155** | **205** | **212** | **221** | **222** |
| A/California/07/2009 | D | I | E | V | T | V | L | L | I | E | K | A | N | P | K | V |
| A/Michigan/45/2015 | E | I | K | M | T | V | I | L | V | D | E | A | S | P | E | V |
| A/Victoria/2570/2019 | E | I | K | V | A | V | I | L | V | D | E | T | S | P | E | V |
| A/Sydney/5/2021 | E | V | K | V | A | I | I | P | V | D | E | T | S | P | E | M |
| A/Victoria/4897/2022 | E | I | K | V | A | I | I | L | V | D | E | T | S | S | E | M |
| **NS Position** | **224** | **225** | **235** | **241** | **242** | **243** | **249** | **262** | **268** |  |  |  |  |  |  |  |
| A/California/07/2009 | R | A | V | A | A | Q | E | T | T |  |  |  |  |  |  |  |
| A/Michigan/45/2015 | G | T | V | T | A | Q | E | T | A |  |  |  |  |  |  |  |
| A/Victoria/2570/2019 | G | T | M | T | T | Q | K | T | A |  |  |  |  |  |  |  |
| A/Sydney/5/2021 | G | T | M | T | T | Q | K | T | A |  |  |  |  |  |  |  |
| A/Victoria/4897/2022 | G | T | M | T | T | K | K | N | A |  |  |  |  |  |  |  |
