# Supplementary material for: Updated A(H1N1)pdm09 influenza virus ferret infection model permits refined antiviral assessment using aerosol inhalation challenge
Source: PLoS Pathog. 2026 Sep 9;22(9):e1014604. doi: 10.1371/journal.ppat.1014604 (PMC13588513; doi:10.1371/journal.ppat.1014604)
Supplement: Supplementary file 3 [file ppat.1014604.s003.docx]

**Supporting Figure 2. Weight loss and temperature of ferrets following intranasal inoculation of ferrets with different A(H1N1)pdm09 viruses.** Ferrets were infected intranasally with 5 log_10_TCID_50_/mL units of A/California/07/2009, A/Michigan/45/2015, A/Victoria/2570/2019, A/Sydney/5/2021 or A/Victoria/4897/2022 virus in 500 µL. At day three or five post infection animals were culled for tissue harvest, n= 4 per group (except A/Sydney/5/21 n=8 at day five). (a) Weight and (b) body temperature changes from baseline were recorded daily. Area under the curve (AUC) analysis was performed to compare the net (c) weight change and (e) temperature change over time between ferret groups. Peak recorded (d) weight loss and (f) temperature rise over the duration of infection were also analysed. Mean and SD are shown for weight and temperature changes, analysis performed by One-way ANOVA Tukey post-hoc test (* p<0.05, ** p<0.01, *** p<0.001).
