# Supplementary material for: Updated A(H1N1)pdm09 influenza virus ferret infection model permits refined antiviral assessment using aerosol inhalation challenge
Source: PLoS Pathog. 2026 Sep 9;22(9):e1014604. doi: 10.1371/journal.ppat.1014604 (PMC13588513; doi:10.1371/journal.ppat.1014604)
Supplement: Supplementary file 4 [file ppat.1014604.s004.docx]

**Supporting Figure 3.** **Influenza A(H1N1)pdm09 virus induced histopathological changes in the lung lobes of ferrets**. Ferrets infected with 5 log_10_TCID_50_/500uL of A/California/07/2009, A/Michigan/45/2015, A/Victoria/2570/2019, A/Sydney/5/2021, or A/Victoria/4897/2022 were culled at day five post intranasal inoculation. Intact trachea and lungs were removed, inflated with formalin for fixation. Representative lung lobe sections embedded in paraffin and sectioned were stained (a) by hematoxylin and eosin, or (b) immunohistochemistry (IHC) with influenza A virus nucleoprotein (IAV NP) antibody and a hematoxylin counterstain, and (c) higher magnification scans (taken from black squares) revealed sites of infection (black arrows). Two images of the same lung section are shown in the top and bottom panel.
