# Supplementary material for: Updated A(H1N1)pdm09 influenza virus ferret infection model permits refined antiviral assessment using aerosol inhalation challenge
Source: PLoS Pathog. 2026 Sep 9;22(9):e1014604. doi: 10.1371/journal.ppat.1014604 (PMC13588513; doi:10.1371/journal.ppat.1014604)
Supplement: Supplementary file 5 [file ppat.1014604.s005.docx]

**Supporting Table 2. Ferret influenza transmission studies, nasal wash virus shedding and seroconversion analysis.**

|  | ***Airborne Exposure*** | ***Contact exposure*** |
| --- | --- | --- |
| ***Transmission events**** | 6/8 | 11/11 |
| ***Donor ferret nasal wash (NW) viral titre at D2 post-exposure (log_10_TCID_50_/mL)*** | 4.2, 4.5 , 4.2, 5.2, 4.7, 5.2, 6.0, 5.2 | 4.7, 5.0, 5.2, 4.2, 4.5, 5.2, 4.5, 4.5, 6.0, 3.7, 4.7 |
| ***Donor ferret NW AUC - resulting in productive transmission event*** | 15.5, 16.0, 18.8, 16.6, 16.3, 19.0 | *Not tested* |
| ***Donor ferret NW AUC - failed transmission event*** | 16.0, 16.3 | *Not tested* |
| ***Day post exposure recipient ferret viral shedding*** | 1, 2, 2, 2, 2, 3, NA | ≤2^#^ (all eleven) |
| ***Recipient ferret duration of viral shedding (days)*** | 6, 6, 6, 7, 7, 7, NA | 5, 5, 5, 5, 5, 5, 5, 5, 7, 7, 7, 7 |
| ***Recipient ferret HAI seroconversion (>40 HAI units)*** | 7/8 | *Not tested* |

*Transmission event defined as detection of infectious virus from NW specimen of recipient ferret

^#^day one post exposure NW was not tested.
