# Supplementary material for: Updated A(H1N1)pdm09 influenza virus ferret infection model permits refined antiviral assessment using aerosol inhalation challenge
Source: PLoS Pathog. 2026 Sep 9;22(9):e1014604. doi: 10.1371/journal.ppat.1014604 (PMC13588513; doi:10.1371/journal.ppat.1014604)
Supplement: Supplementary file 6 [file ppat.1014604.s006.docx]

**Supporting Table 3. Virus amino acid variants of sequenced ferret nasal turbinates and nasal washes from A/Sydney/5/2021 oseltamivir antiviral efficacy study.**

| ***Inoculation Method*** | ***Tissue sample (Day 5 p.i.)*** | ***Ferret #*** | ***Treatment*** | ***Amino acid variants >10% frequency*** | | |
| --- | --- | --- | --- | --- | --- | --- |
|  |  |  |  |  | ***NA*** |  |
| A/Sydney/5/2021 virus stock | | | | - | - |  |
| *Aerosol exposure* | *Nasal turbinate* | *1* | *Mock* |  | ***-*** |  |
|  |  | *2* | *Mock* | ***-*** | 20% V234L |  |
|  |  | *3* | *Mock* |  | ***-*** |  |
|  |  | *5* | *Pre-tx* |  | ***-*** |  |
|  |  | *10* | *Post-tx* | ***-*** | **9% H275Y** |  |
|  | *Nasal wash* | *1* | *Mock* |  | ***-*** |  |
|  |  | *4* | *Mock* |  | ***-*** |  |
|  |  | *5* | *Pre-tx* |  | ***-*** |  |
|  |  | *10* | *Post-tx* |  | **41% H275Y** |  |
|  |  | *12* | *Post-tx* |  | **13% H275Y** |  |
| *Intranasal*  *Inoculation* | *Nasal turbinate* | *13* | *Mock* | ***-*** | 18% V234L |  |
|  |  | *15* | *Mock* |  | ***-*** |  |
|  |  | *17* | *Pre-tx* | - | - |  |
|  |  | *18* | *Pre-tx* |  | - |  |
|  |  | *23* | *Post-tx* |  | - |  |
|  |  | *24* | *Post-tx* |  | - |  |
|  | *Nasal wash* | *15* | *Mock* |  | - |  |
|  |  | *17* | *Pre-tx* |  | **20% H275Y** |  |
|  |  | *21* | *Post-tx* |  | **29% H275Y** |  |
|  |  | *23* | *Post-tx* |  | **11% H275Y** |  |
|  |  | *24* | *Post-tx* |  | **-** |  |
