# Supplementary material for: Updated A(H1N1)pdm09 influenza virus ferret infection model permits refined antiviral assessment using aerosol inhalation challenge
Source: PLoS Pathog. 2026 Sep 9;22(9):e1014604. doi: 10.1371/journal.ppat.1014604 (PMC13588513; doi:10.1371/journal.ppat.1014604)
Supplement: Supplementary file 7 [file ppat.1014604.s007.docx]

**Supporting Figure 4. Comparison of infectious virus and RNA titres in nasal wash samples while assessing oseltamivir antiviral efficacy to reduce A(H1N1)pdm09 viral load in the respiratory tract of ferrets following aerosol exposure or intranasal inoculation.** Ferrets were inoculated by the aerosol or intranasal (1 mL) route with A(H1N1)pdm09 A/Sydney/5/2021 virus at 50 PFU. Oseltamivir phosphate (5 mg/kg) or sugar water (mock: black) as a control was administered twice daily, either one day prior to inoculation until day four (pre-tx: pink), or one day post inoculation until day four (post-tx: green), n = 4 per group. Viral titres in nasal wash (NW) specimens were determined daily following either aerosol exposure or intranasal inoculation by standard plaque assay (Infectious titre) or quantitative PCR of the viral matrix gene (Viral RNA titre) on day two and five only. Mean and SD, compared across each group by two-way ANOVA Tukey pos t-hoc test (* p<0.05, ** p<0.01, *** p<0.001, **** p<0.0001). Open symbols indicate the detection of NA-H275Y amino acid change at various frequencies in NW or nasal turbinates at day 5 (□ - 6-20%, Δ - 21-35%, ○ - 36-50%).
