# Supplementary material for: Updated A(H1N1)pdm09 influenza virus ferret infection model permits refined antiviral assessment using aerosol inhalation challenge
Source: PLoS Pathog. 2026 Sep 9;22(9):e1014604. doi: 10.1371/journal.ppat.1014604 (PMC13588513; doi:10.1371/journal.ppat.1014604)
Supplement: Supplementary file 8 [file ppat.1014604.s008.docx]

**Supporting Figure 5. Viral load in ocular and gastrointestinal sites of ferrets following A(H1N1)pdm09 virus aerosol exposure or intranasal inoculation to evaluate oseltamivir antiviral efficacy**. Ferrets were inoculated by the aerosol or intranasal route with A(H1N1)pdm09 A/Sydney/5/2021 virus at 50 PFU. Oseltamivir phosphate (5 mg/kg) or sugar water as a control was administered twice daily, either one day prior to infection to day three (Pre-tx), or one day post infection until day four (Post-tx), n = 4 per group. Viral titres in daily conjunctiva wash (CW) were determined following either (a) aerosol exposure or (c) intranasal inoculation by plaque assay. Additionally, daily rectal swabs (RS) were titrated following (e) aerosol and (g) intranasal infection. At day five post infection (b,d) conjunctiva and (f,h) intestine tissue was harvested for virus titration. Mean and SD are compared across each group. Tissue viral load was compared by Kruskal-Wallis with Dunn’s multiple comparison post-hoc test (* p<0.05, ** p<0.01).
