# Supplementary material for: Updated A(H1N1)pdm09 influenza virus ferret infection model permits refined antiviral assessment using aerosol inhalation challenge
Source: PLoS Pathog. 2026 Sep 9;22(9):e1014604. doi: 10.1371/journal.ppat.1014604 (PMC13588513; doi:10.1371/journal.ppat.1014604)
Supplement: Supplementary file 9 [file ppat.1014604.s009.docx]

**Supporting Table 4. Influenza A(H1N1)pdm09 A/Sydney/5/2021 neuraminidase inhibition (NAI) assay IC_50_ assessment.**

| ***NAI*** | ***Mean IC_50_ (+SD) nM (n=3)*** | | |
| --- | --- | --- | --- |
|  | ***A/Sydney/5/2021*** | ***A/Perth/82/2015 (WT Susceptible Control)*** | ***A/Osaka/180/2009 (NA-H275Y OST Resistant Control)*** |
| ***Oseltamivir*** | 1.0 (0.6) | 1.2 (1.0) | 496.0 (390) |
| ***Peramivir*** | 0.2 (0.1) | 0.2 (0.1) | 56.6 (26) |
| ***Zanamivir*** | 0.7 (0.4) | 0.6 (0.3) | 0.6 (0.3) |
| ***Laninamivir*** | 1.0 (0.5) | 0.8 (0.4) | 1.1 (0.4) |
